# Physics-Informed Gaussian Process Inference of Liquid Structure from Scattering Data

**DOI:** 10.1021/acs.jpcb.5c05024

**Published:** 2025-10-31

**Authors:** Harry Winston Sullivan, Matej Cervenka, Brennon L. Shanks, Michael P. Hoepfner

**Affiliations:** † Department of Chemical Engineering and Material Science, 41485 University of Minnesota - Twin Cities, Minneapolis, Minnesota 55455, United States; ‡ Institute of Organic Chemistry and Biochemistry of the Czech Academy of Sciences, Flemingovo nám. 2, 166 10 Prague 6, Czech Republic; § Department of Chemical Engineering, 7060 University of Utah, Salt Lake City, Utah 84112-9203, United States

## Abstract

We present a nonparametric Bayesian framework to infer radial distribution functions from experimental scattering measurements with uncertainty quantification using nonstationary Gaussian processes. The Gaussian process prior mean and kernel functions are designed to mitigate well-known numerical challenges with the Fourier transform, including discrete measurement binning and detector windowing, while encoding fundamental yet minimal physical knowledge of the liquid structure. We demonstrate uncertainty propagation of the Gaussian process posterior to unmeasured quantities of interest. Experimental radial distribution functions of liquid argon and water with uncertainty quantification are provided as both a proof of principle for the method and a benchmark for molecular models.

## Graphical abstract

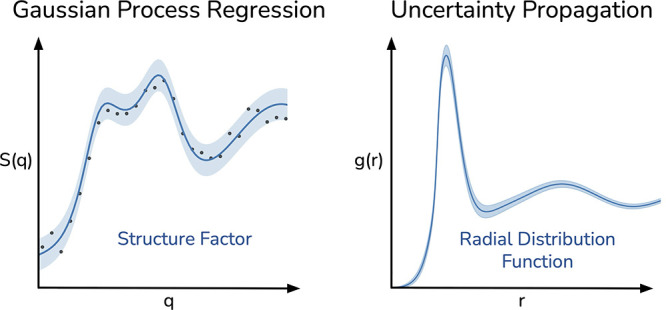

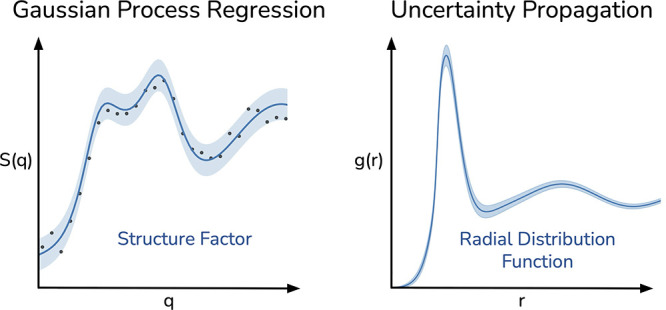

## Introduction

The radial distribution function (RDF), which characterizes the spatial arrangement of atoms, is a cornerstone in liquid state theory and serves as a vital benchmark for molecular simulations. Our understanding of the liquid state relies heavily on established theoretical relationships that link the RDF to thermodynamic properties and interatomic forces. These include the Ornstein–Zernike relation,^1^ Henderson’s inverse theorem,^2^ the Born–Bogilubov–Green–Kirkwood–Yvon hierarchy,^3^ and Kirkwood-Buff integrals,^4^ among others. Despite these profound and intricate connections, structure is often relegated to a validation step in molecular modeling, with preference typically given to training force field parameters using macroscopic thermodynamic data^5^ or to interatomic potentials computed from quantum mechanical methods.^6^ While both approaches can yield models that accurately reproduce the thermophysical properties of fluids, they often struggle to fully capture structural features observed in experiments.^7–9^ We argue that to more closely align molecular models with the principles of statistical mechanics, greater emphasis should be placed on experimentally derived RDFs in force field optimization and design.

In X-ray and neutron scattering experiments, the observed quantity is the momentum-space static structure factor, from which RDFs are subsequently inferred.^10^ For single-atom systems, the static structure factor is related to the RDF, or *g*(*r*), via a Fourier transform with radial symmetry,

$$S(q)-1=4\pi \rho \int_{0}^{\infty }(g(r)-1)\frac{\sin(qr)}{qr}r^{2}dr$$ 1

where *q* is the momentum transfer and ρ is the atomic number density. In mixtures or molecular liquids, the total structure factor, *F*(*q*), can be expressed as a combination of site–site partial structure factors, *S*_*ij*_, between atoms *i*, *j,* such that,

$$F\left(q\right)=\underset{i\geq j}{\sum }\left[2-\delta_{ij}\right]w_{ij}S_{ij}\left(q\right)$$ 2

where *w*_*ij*_ is a (possibly *q-*dependent in the case of X-rays) weighting factor depending on the scattering length density and atomic concentration of the *i*, *j* pair, and δ_*ij*_ is the Kronecker delta. This linear system, known as the Faber–Ziman decomposition,^11^ is ill-posed when the number of measured total structure factors is fewer than the number of unique site–site partial structure factors, which for a system with *N* distinct atom types has *N*(*N* + 1)/2 unique *S*_*ij*_ terms. To constrain this underdetermined linear system, scattering measurements can be performed on isotopologues (systems differing only by isotopic substitutions), which alter the scattering length densities without changing the underlying structure. However, in practice, obtaining a sufficient number of isotopologue measurements is often prohibitively expensive in terms of both experimental time and the cost of purified isotopes. As a result, solutions to the Faber–Ziman decomposition have historically been approximated using iterative molecular simulation methods to close the linear system with simulated structure data, such as reverse Monte Carlo (RMC)^12^ and empirical potential structure refinement (EPSR).^13^

Assuming that the partial structure factors are known, they can then be Fourier transformed with eq 1 to obtain real-space site–site pair distribution functions, *g*_*i*,*j*_(*r*), which quantify the atomic density of type *i* within a spherical shell around any atom of type *j*. The *g*_*i*,*j*_(*r*) describes the relative likelihood of finding a neighboring atom at distance *r*; in liquids, it goes to zero at small *r* due to atom–atom impenetrability, exhibits oscillations that reflect local structure, and approaches unity at large *r* where correlations vanish.Eqs 1 and 2 are conceptually appealing, but their practical implementation faces several challenges. First, the finite size of individual neutron detectors constrains structure factor measurements to discrete momentum transfer values, Δ*q* = *q*_*i*_ – *q*_*i*–1_, which, according to the Peterson-Middleton sampling theorem,^14^ can result in aliasing if the sampling efficiency is <1. Second, finite detector coverage windows the measurement to a range between some *q*_min_ and *q*_max_, preventing the evaluation of the full integral specified in eq 1. Windowing can introduce truncation artifacts (ripples), reduce the real-space resolution, and, when a smooth windowing correction is applied, artificially broaden the RDF peaks. Finally, measurement uncertainty of neutron counts and momentum transfer positions (i.e., time-of-flight uncertainty) introduces noise that can corrupt the underlying signal.^15,16^

In practice, a discrete radial Fourier transform (rFT) must be computed over *N* uncertain observations,

$$g\left(r\right)\approx 1+\frac{1}{2\pi^{2}\rho }\overset{N}{\underset{i=1}{\sum }}\frac{1}{2}\left(S\left(q_{i-1}\right)\frac{\sin\left(q_{i-1}r\right)}{q_{i-1}r}q_{i-1}^{2}-S\left(q_{i}\right)\frac{\sin\left(q_{i}r\right)}{q_{i}r}q_{i}^{2}\right)\Delta q$$ 3

where the sum is from some nonzero *q*_min_ to some finite *q*_max_ = *N*Δ*q*. The key problem is that, depending on the degree of undersampling and the choice of window function, the discrete Fourier transform can systematically distort the predicted RDF relative to the ground truth. These distortions, in turn, increase the uncertainty in the inferred fluid structure. This uncertainty may partially explain why scattering data are not more widely used as an optimization target in force field design.

The most well-studied problem in prior literature is addressing the *q*_max_ cutoff using so-called modification functions.^17^ The essential idea here is to smoothly transition the structure factor from a data-dominated section (as measured by the neutron/X-ray detector) to a model-driven section (dictated by prior physical knowledge of the structure factor). Modification functions are designed to force the contribution of the experimental data to 0 near *q*_max_, effectively nullifying any features in the data and strictly relying on the physical model alone. Usually, the data are transitioned into a Poisson point process ideal gas model (i.e., *S*_Ideal_(*q*) = 1).^18^ Mathematically, this modifies the integral of eq 1 into,

$$g(r)=1+\frac{1}{2\pi^{2}\rho }\int_{0}^{\infty }(S(q)-1)M(q)\frac{\sin(qr)}{qr}q^{2}dq$$ 4

where *M*(*q*) is the *q*-dependent modification function. Common choices for the modification function are the first Bessel function,^19^ second Bessel function,^20,21^ cosine cutoff,^22^ and dynamic functions.^23^ However, as pointed out by Proctor et al.,^17^ eq 4 is an approximate Bayesian predictive model where the modification function transitions into a prior *S*(*q*) model. To see this, one can rewrite eq 4 in the following way,

$$=1+\frac{1}{2\pi^{2}\rho }\int_{0}^{\infty }(\underset{\mathrm{Data}\;\mathrm{Driven}\;\mathrm{Predictive}}{\underset{︸}{\left(S(q)-1)M(q\right)}}+\underset{\mathrm{Model}\;\mathrm{Driven}\;\mathrm{Predictive}}{\underset{︸}{\left(S_{\mathrm{Ideal}}(q)-1)(1-M(q)\right)}})\frac{\sin(qr)}{qr}q^{2}dq$$ 5

where we have split the two contributions of the integrand into “data-driven” and “model-driven” parts, regulated by the modification function. Here, the *M*(*q*) is viewed as a discrete posterior probability mass, meaning that all we have done is expressed the structure factor as a weighted mixture of two outcomes, either data or model, at each *q* value. An extensive analysis of commonly used modification functions can be found in ref 17.

While using prior information to constrain the space of possible RDFs is a valuable idea, the formulation above does not naturally support uncertainty quantification in the RDF predictions within a probabilistic framework. Prior studies have attempted to estimate uncertainty in scattering data by averaging RDF predictions across multiple experiments and computing standard deviations,^24^ propagating experimental structure factor errors through the Fourier transform,^25^ or combining both methods.^23^ The primary limitation of simply comparing different data sets or analysis methods is that such estimates become unreliable if all sources share a common systematic error. Similarly, the Fourier transform error propagation method produces the largest uncertainties at small *r*, precisely where theories of interatomic forces dictate that the RDF must vanish. This results in unrealistic uncertainty estimates. A more rigorous approach has been introduced in which Bayesian uncertainty quantification is applied to the interatomic potential using experimentally derived RDFs as observations.^26^ However, this parametric approach relies on a predefined functional form for the potential (for example, a Lennard–Jones or Mie potential), inherently constraining the model and introducing unnecessary bias. What is needed, therefore, is a probabilistic framework that can incorporate known physical features of the RDF, such as short-range exclusion and long-range decay, while remaining flexible enough to avoid biases imposed by assumed potential forms or molecular simulation models.

We propose that a mathematically rigorous version of the RDF posterior satisfying these requirements can be computed through the use of Bayesian inference on the experimental structure factor directly. Specifically, by placing a Gaussian process (GP) prior over the experimental structure factor, multiplying it with an appropriate likelihood function, and finally computing the rFT over the resulting *q*-space posterior distribution, we obtain a Bayesian posterior distribution on the RDF. The use of a prior regularizes the infinite set of possible functions that could fit the finite observed data set, while the likelihood serves as a data fit penalty. The resultant posterior distribution on the RDF represents a direct uncertainty quantification over the real-space structure, given the momentum space scattering observations.

GPs have been used extensively in solving ill-posed inverse problems in computational chemistry,^6^ including Fourier analysis of noisy and truncated signals, which plagues the scattering problem.^27^ A GP-based approach was recently developed to analyze small-angle neutron scattering data, demonstrating that GP predictions can optimize neutron beamtime usage and increase experimental throughput without compromising data quality.^28^ Furthermore, GPs naturally resolve many of the current challenges of scattering analysis cited earlier. For example, they can infer the structure factor on a continuum of momentum values with a domain consistent with the rFT (from *q* = 0 to lim_*q*→*∞*_ *S*(*q*)). As long as the GP mean and kernel selection are physics-informed and flexible enough to represent the data, Bayesian inference will be robust up to available experiments and our theoretical understanding of the structure. Such a framework is more elegant and satisfactory than, say, neural networks or other black-box machine learning tools that often ignore expert knowledge and do not have uncertainty quantification built into their mathematical formalism. The GP framework, therefore, supports inference while maintaining transparency and expert interpretability.

In this study, we present a probabilistic machine learning framework to estimate total or partial RDFs with uncertainty quantification from background and inelastic corrected scattering data using nonstationary GP regression. We show how nonstationary GPs with a physics-informed mean and kernel conditioned on experimental scattering data enable the complete reconstruction of the atomic structure from both simulation and experimentally derived total structure factors. The mean and kernel selection reflect simple and indisputable properties of the RDF, including the correct limiting behaviors for realistic bulk fluids (lim_*r*→0_ *g*(*r*) = 0, lim_*r*→*∞*_ *g*(*r*) = 1), continuity and differentiability, and the presence of bonded and nonbonded contributions.

As test cases for the nonstationary GP model, we performed RDF inference for a simple liquid (argon) and a complex liquid (water) from structure factors derived from both simulation and experiment. All inferred RDF distributions are free from spurious Fourier artifacts and preserve tailing behaviors as dictated by the nonstationary kernel. For liquid argon, we find that the nonstationary GP prediction shows near-perfect agreement with a gold-standard neutron scattering analysis from Yarnell.^29^ For water structure obtained from a classical water model with flexible bonds, the nonstationary GP regression reconstructs the ground truth data even with significant noise introduced to the structure factor signal. Once the nonstationary GP was validated, we investigated an X-ray scattering data set of liquid water^23^ to obtain a novel interpretation of the oxygen-oxygen RDF distribution that can be compared with molecular models of water.

At first glance, it might seem relatively uninteresting to perform Bayesian inference over the RDF prediction. However, this type of computation can serve as a key result for validation of molecular dynamics simulation^23,30^ and help unlock emerging methods in computational chemical physics. For instance, access to an RDF distribution as a GP could serve as a link between the increasingly popular Gaussian approximation potential (GAP) framework^31^ and experimental scattering data. Additionally, force field optimization algorithms such as structure optimized potential refinement (SOPR),^32,33^ which models the interatomic potential as a GP, can now propagate uncertainty directly from experimental observation into the estimation of interatomic potentials. The same is true of parametric Bayesian force field inference, which can be employed with the methods presented here to estimate how well a given molecular model represents complex experimental data. Finally, as Bayesian interpretations become more frequently integrated into chemical physics, these approaches will be necessary to understand model uncertainties with respect to experiments, a critical step of the scientific method that has been largely under-reported in the existing literature due to a lack of rigorous approaches to estimate uncertainty in complex experimental observables. This framework, therefore, allows us to leverage all available data without throwing away established physical knowledge accumulated through generations of scientific discovery (Figure 1).

**Figure 1:**
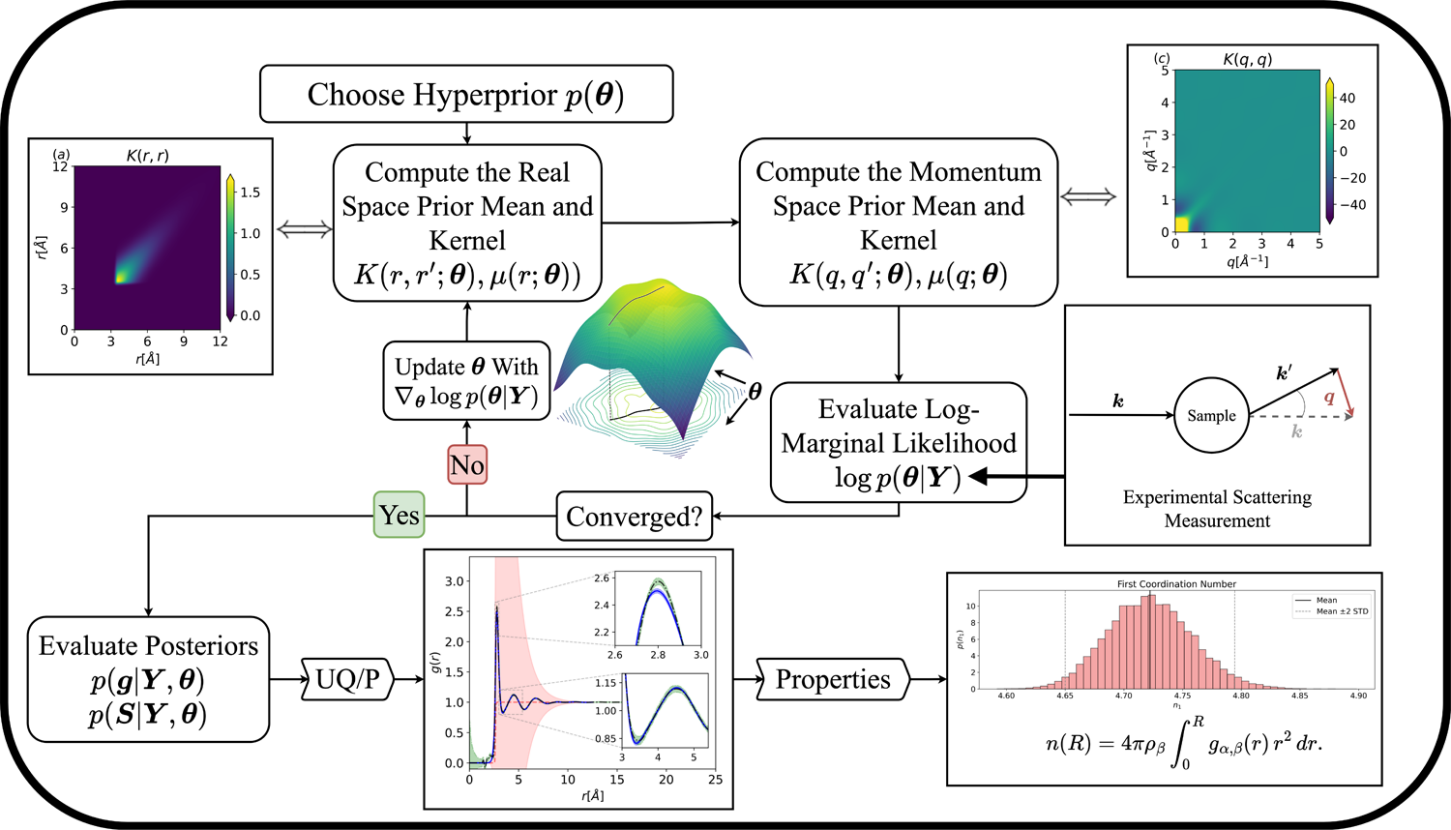
A flowchart corresponding to the GPFT algorithm applied to scattering data.

## Theory and Methods

Consider a structure factor, which is unknown to us, that lives within a *distribution* of possible functions that are known to obey specific physical characteristics. We can write this mathematically,[Fn fn1] in the context of a GP by stating that any evaluation (or set of evaluations) of the unknown function, *S*(*q*), is distributed as a multivariate Gaussian,

$$S\left(q\right)∼GP\left(\mu \left(q\right),K\left(q,q^{\prime }\right)\right)$$ 6

where the mean, μ­(q), represents the a priori expected value of the function at each point in the input space, and the covariance (or kernel) function, *K*(*q*, *q*′), represents the relatedness of the output quantities with respect to the process inputs. The mean and kernel constrain the set of possible functions that could represent the experimental observation to those consistent with physical intuition. The nonstationary behavior of such a GP refers to the fact that the Fourier transform of the true structure factor has limiting behavior with certainty (i.e., lim_*r*→0_ *g*(*r*) = 0, lim_*r*→*∞*_ *g*(*r*) = 1), meaning that the functional distribution has covariances that change with respect to its inputs.^34^

A physics-informed GP model enables Bayesian inference of a structure factor posterior distribution conditioned on the experimental scattering data. This posterior reflects uncertainties from both known physical principles and experimental observations, offering a reliable and robust estimation of the uncertainty in the liquid structure. Finally, due to the linearity of the rFT, this uncertainty can be propagated into the prediction of the RDF and subsequently compared to molecular simulation predictions. This rigorous representation of our current knowledge of the liquid structure serves as a powerful validation tool for molecular models and helps to pinpoint key measurements necessary to resolve gaps in our understanding of the organization of molecules in liquids.

### The GP Model

Bayes’ theorem provides a natural route to compute the structure factor posterior, *p*(***S*** | ***Y***, **θ**), according to Bayes theorem,

$$p(S|Y,\theta )=\frac{p(Y|S,\theta )p(S|\theta )}{p(Y|\theta )}=p(Y|S,\theta )p(S|\theta )[\int p(Y|S,\theta )p(S|\theta )dS{]}^{-1}$$ 7

where *p*(***S*** |**θ**), *p*(***Y*** | ***S***, **θ**), and *p*(***Y*** |**θ**) are the prior, likelihood, and model evidence, respectively. Here, ***Y*** is the set of experimental observations, ***S*** is the value of the structure factor due to some inducing vector, and **θ** is the set of GP hyperparameters.

The *GP prior* over the inducing index vector of momentum transfer values ***q*** is defined by a mean, μ­(***q***) = **μ**_***q***_, and a kernel, *K*(***q***, ***q***) = ***K̂***_***q***, ***q***_, function, which, when evaluated on the index set, produces a vector and a matrix, respectively,

$$p(S|\theta )=(2\pi {)}^{-d/2}\det|{\hat{K}}_{q,q}{|}^{-1/2}\exp((S-\mu_{q}{)}^{T}{\hat{K}}_{q,q}^{-1}(S-\mu_{q}))$$ 8

where the determinant is required due to the nondiagonal covariance between the latent function values. Note that this quantity is just a GP representation of the structure factor distribution before seeing any data.

The *likelihood* is the probability that the observed data are generated by a particular instance of ***S***. Assuming that the structure factor has approximately spatially uncorrelated and constant Gaussian noise (which is the case for reactor source neutron scattering^10^), an appropriate likelihood is a homoscedastic normal distribution,

$$p(Y|S,\theta )=(2\pi \omega^{2}{)}^{-d/2}\exp((S-Y{)}^{T}(\omega^{2}\hat{I}{)}^{-1}(S-Y))$$ 9

where ω is a noise parameter, *d* is the number of observed data points, and ***Î*** is the identity matrix. Modified versions of this expression would be required for systems with significant heteroscedastic noise, such as spallation source neutron instruments. In this case, the likelihood can be generalized by replacing the scalar variance ω^2^ with a function ω^2^(***q***) varying along the diagonal of the covariance matrix while remaining jointly Gaussian.^35^ In general, as long as the observations are independent, the likelihood can represent any noise distribution, underscoring the flexibility of the Bayesian framework.^36^ Note that the dependence of these expressions on hyperparameters **θ** stems from the underlying kernel and mean functions used to evaluate ***S***.

Finally, the conjugacy of Gaussian distributions for both the prior and likelihood enables analytical integration of the *model evidence* (also known as the marginal likelihood),^37,38^ and upon taking the log to improve numerical stability, gives,

$$\log\;p(Y|\theta )=-\frac{1}{2}(Y-\mu_{q}{)}^{T}({\hat{K}}_{q,q}+\omega^{2}\hat{I}{)}^{-1}(Y-\mu_{q})-\frac{1}{2}\log\;\det|{\hat{K}}_{q,q}+\omega^{2}\hat{I}|-\frac{d}{2}\log\;2\pi$$ 10

Combining these expressions into eq 7, the posterior distribution over the latent function ***S*** evaluated at some *m-*sized index vector ***q*** * is then,

$$p(S|Y,\theta )=(2\pi {)}^{-m/2}\det|{\hat{\Sigma }}_{\mathrm{Post}}{|}^{-1/2}\exp((S-\mu_{\mathrm{Post}}{)}^{T}{\hat{\Sigma }}_{\mathrm{Post}}^{-1}(S-\mu_{\mathrm{Post}}))$$ 11

where the posterior mean and variance are given by,

$$\mu_{\mathrm{Post}}=\mu_{q*}+{\hat{K}}_{q*,q}({\hat{K}}_{q,q}+\omega^{2}\hat{I}{)}^{-1}(Y-\mu_{q})$$ 12

$${\hat{\Sigma }}_{\mathrm{Post}}={\hat{K}}_{q*,q*}-{\hat{K}}_{q*,q}({\hat{K}}_{q,q}+\omega^{2}\hat{I}{)}^{-1}{\hat{K}}_{q,q*}$$ 13

### Posterior Estimation of the RDF

The next step is to propagate uncertainty from the structure factor distribution into real space. By the fluctuation–dissipation theorem, the RDF is related to the structure factor through a 3D Fourier transform, which, assuming spherical symmetry, can be written as the well-known rFT, which we denote $\tilde{H}$ ,

$${\tilde{H}}_{q}[f(q)]=\frac{1}{2\pi^{2}\rho }\int_{0}^{\infty }f(q)\frac{\sin(qr)}{qr}q^{2}dq,{\tilde{H}}_{r}^{-1}[f(r)]=4\pi \rho \int_{0}^{\infty }f(r)\frac{\sin(qr)}{qr}r^{2}dr$$ 14

which maps a function of *r* to a function of *q*. Notably, the inverse of the rFT proportional to the operator is itself up to a proportionality constant ( $\tilde{H}=8\pi^{3}\rho^{2}{\tilde{H}}^{-1}$ , see Supporting Information Section S2 for details) and is linear with respect to the input function. This operator is related to the Hankel transform.^39^ The RDF structure factor relationship is then,

$$S(q)=1+{\tilde{H}}_{r}^{-1}[g(r)-1],g(r)=1+{\tilde{H}}_{q}[S(q)-1]$$ 15

At first glance, it may seem unclear how to apply eq 15 to a distribution of structure factors; however, the normality of the GP, in tandem with the linearity of the operator, can alleviate nearly all of the difficulty since the resulting distribution is trivially Gaussian. This nice property is due to the well-known fact that the linear transformation of a finite-dimensional Gaussian distribution is again Gaussian,

$$z∼N(\mu ,\hat{\Sigma })$$ 16

$$\Rightarrow \hat{A}z∼N(\hat{A}\mu ,\hat{A}\hat{\Sigma }{\hat{A}}^{T})$$ 17

where ***Â*** is a linear operator acting on a finite-dimensional vector ***z***. Assuming that the linear operator is bounded and densely defined, the same property holds for GPs.^40^ This approach is often leveraged in the analysis of partial differential equations^41^ and can be applied to the rFT integral operator defined in eq 15.

Eq 17 has important implications for relating kernels between the Fourier duals of momentum and real space. For example, we can now construct new kernels in the Fourier dual space by applying the linear rFT operator,

$$K(r,r^{\prime })=\mathrm{cov}(g(r),g(r^{\prime })={\tilde{H}}_{q}[{\tilde{H}}_{q\prime }[K(q,q^{\prime })]]$$ 18

$$K(r,q^{\prime })=\mathrm{cov}(g(r),S(q^{\prime }))={\tilde{H}}_{q}[K(q,q^{\prime })]$$ 19

$$K(q,r^{\prime })=\mathrm{cov}(S(q),g(r^{\prime }))={\tilde{H}}_{r}^{-1}[K(r,r^{\prime })]$$ 20

$$K(q,q^{\prime })=\mathrm{cov}(S(q),S(q^{\prime }))={\tilde{H}}_{r}^{-1}[{\tilde{H}}_{r\prime }^{-1}[K(r,r^{\prime })]]$$ 21

In essence, the RDF posterior distribution reflects correlations between observed data in *q*-space projected into *r*-space, giving the overall probability of the RDF ***g*** evaluated on an *n-*sized index vector ***r*** as,

p(g|Y,θ)=(2π)n/2det|Σ^Post,RDF|−1/2exp((g−μPost,RDF)TΣ^Post−1(g−μPost,RDF)) 22

with posterior mean and variance,

$$\mu_{\mathrm{Post},\mathrm{RDF}}=\mu_{r}+{\hat{K}}_{r,q}({\hat{K}}_{q,q}+\omega^{2}\hat{I}{)}^{-1}(Y-\mu_{q})$$ 23

$${\hat{\Sigma }}_{\mathrm{Post},\mathrm{RDF}}={\hat{K}}_{r,r}-{\hat{K}}_{r,q}({\hat{K}}_{q,q}+\omega^{2}\hat{I}{)}^{-1}{\hat{K}}_{q,r}$$ 24

where **μ**_***r***_ is just ${\tilde{H}}_{q}[\mu (q)-1]$ evaluated at ***r***. Formally, these expressions may also be obtained by application of the rFT ${\tilde{H}}_{q}$ operator to the *S*(*q*) posterior evaluated at a single inducing point *q*.

While the above method works in theory, not all of the integrals are analytically tractable and conducive to pen and paper computation. Indeed, it is more practical to use an approximate operator, $\tilde{H}$ , computed with simple numerical quadrature,

$${\overset{\sim }{H}}_{q}[f(q)]\approx \overset{N}{\underset{i=1}{\sum }}\frac{1}{4\pi^{2}\rho }\;\left(f(q_{i-1})\frac{\sin(q_{i-1}r)}{q_{i-1}r}q_{i-1}^{2}-f(q_{i})\frac{\sin(q_{i}r)}{q_{i}r}q_{i}^{2}\right)\Delta q={\overset{\sim }{H}}_{q}[f(q)]$$ 25

where the grid of *q* values is over the range of the integral. Note that the approximate operator $\tilde{H}$ acts on a discretized grid of function values *f*(***q***) and produces a single number which corresponds to the implicit radial argument *r*. The choice of grid spacing can affect the resulting RDFs and uncertainty predictions; therefore, care must be taken to ensure the grid resolves all relevant features of the kernel and mean functions used. Details on the exact grid spacing and limits used are provided in the Supporting Information. This approximate rFT operator retains linearity while generalizing to custom GP prior means and kernels that do not have analytical rFTs. This strategy holds connections with typical Bayesian quadrature techniques.^42^ The inverse is discretized similarly to an alternate prefactor.

### Designing a GP for Liquid Structure Factors

The crux of designing any GP model is choosing an appropriate prior, which for a GP is fully specified by its mean and kernel functions and their corresponding hyperparameters. This step is also the most critical for enforcing physical behaviors and constraints in the GP regression.

Because physical correlations are less transparent in momentum space, it is more intuitive to impose constraints directly on the real-space RDF, where structural features are more easily interpreted and well-understood. Given a real-space mean μ­(*r*) and kernel *K*(*r*, *r′*), we can then perform an rFT using one of the techniques from the previous section to obtain *K*(*q*, *q′*) as well as the log marginal likelihood in eq 10. Past work has shown that capturing the limiting behaviors correctly can greatly improve the transform procedure.^17^ Therefore, it is crucial to ensure proper boundary behaviors in the GP prior. We know there must be an excluded volume, as well as a trend toward 1 at the limit, to preserve the overall density of the fluid. Mathematically, these boundary conditions are expressed as,

$$\begin{array}{rl} \underset{r\to 0}{\lim}\;g\left(r\right)=0 & \underset{r\to \infty }{\lim}\;g\left(r\right)=1 \end{array}$$ 26

which can be incorporated into the GP model directly using a nonstationary kernel.

#### Nonstationary Kernel Selection

Unlike stationary kernels, which assume that the covariance only depends on the distance between inducing input locations (*k*(*r*, *r′*) = *k*(|*r* – *r′*|)), nonstationary kernels allow the covariance to change across different regions of the input space. A cleverly designed nonstationary kernel can improve the model’s predictive power by ensuring that the GP adheres to known physical constraints. To see why this is the case, consider that when the kernel evaluation approaches zero, the GP distribution tends toward the mean (c.f. eqs 12 and 13). This provides a natural strategy for enforcing the boundary conditions: force the mean to a known limiting behavior while forcing the covariance to vanish. Specifically, the limits we are after are,

$$\begin{array}{ccc} \lim_{r\;\mathrm{or}\;r^{\prime }\to 0\;\mathrm{or}\;\infty }\;K(r,r^{\prime })=0, & \lim_{r\to 0}\;\mu (r)=0, & \lim_{r\to \infty }\;\mu (r)=1 \end{array}$$ 27

ensuring that the kernel captures localized variations away from the boundaries (*r* = 0 and *r* →*∞*), while the mean function encodes the global boundary behaviors.

Although the RDF is technically a map from $R^{+}$ to $R^{+}$ , the GP itself is not restricted to this domain. To account for this, we impose symmetry with respect to *r* = 0,

$$K_{\mathrm{Sym.}}\left(r,r^{\prime }\right)=K\left(r,r^{\prime }\right)+K\left(-r,r^{\prime }\right)$$ 28

Symmetrizing the kernel prevents artificial asymmetries in the model and ensures that the process behaves consistently across the full input space (for further details on improving numerical stability of kernel calculations, see Supporting Information Section S3 ). Additionally, we know that *g*(*r*) must be continuous and differentiable, as it is required to belong to the radially symmetric Schwartz space to be Fourier transformable.[Fn fn2] Therefore, we based our kernel on the widely used squared exponential kernel, but with nonstationary behavior introduced through *r*-dependent length scale l(r) and a width scale σ­(*r*) functions. The kernel of this type is known as the Gibbs kernel,^43^ which is highly flexible and allows for spatially varying properties,

$$K\left(r,r^{\prime }\right)=\sigma \left(r\right)\sigma \left(r^{\prime }\right)\sqrt{\frac{2l\left(r\right)l\left(r^{\prime }\right)}{l\left(r\right)+l\left(r^{\prime }\right)}}\exp\left(\frac{{-\left(r-r^{\prime }\right)}^{2}}{l\left(r\right)+l\left(r^{\prime }\right)}\right)$$ 29

The flexibility of the Gibbs kernel makes it particularly well-suited for systems where the properties of the process change over space in a known way. By parametrizing σ­(*r*) and l(r) using a chosen functional form, we can further incorporate known behaviors and enhance generalizability. Following the strategy outlined above, we aim to embed as much physically relevant behavior as possible into μ­(*r*), while selecting σ­(*r*) and l(r) to account for deviations from the mean.

In the fluid structures of concern to this work, it is atypical to see large length scale changes as a function of *r* (with the exception of the bonded vs nonbonded structure handled in the mean). This allows us to choose l(r) to be a constant. The limiting behavior of the kernel at large or small inputs is then encoded within σ­(*r*). By ensuring that σ­(*r*) tends to zero as *r* tends to 0, *∞* the kernel will satisfy eq 27. Simple functional forms that satisfy these constraints are a constant length scale function and a decaying sigmoid for the width function

$$\begin{array}{cc} l(r)=l, & \sigma (r)=\frac{\mathrm{Max}\times \exp(\mathrm{Decay}\times \mathrm{Loc})}{1+\exp(-\mathrm{Slope}\times (r-\mathrm{Loc}))}\exp(-r\times \mathrm{Decay})) \end{array}$$ 30

where the hyperparameters Max, Decay, Loc, and Slope control the height, decay rate, peak location, and sharpness of the peak in the sigmoid, respectively.

The presented phenomenological kernel represents an Occam’s razor strategy to kernel design. However, although this kernel satisfies the relevant physical constraints, alternative functional forms with comparable properties and varying levels of rigor are certainly possible. In principle, both l(r) and σ­(*r*) could be derived from first principles, modeled as latent functions (e.g., GPs with their own mean and kernel structures), or selected phenomenologically, as we do here. Conveniently, the Bayesian GP framework provides a principled way to compare kernels through the computation of Bayes factors, which are ratios of their respective marginal likelihoods, $\mathrm{BF}=\frac{p(y|K_{1})}{p(y|K_{2})}$ . The higher the value of the Bayes factor, the more the data support *K*_1_. To compute the marginal likelihood for a candidate kernel, hierarchical inference over the kernel hyperparameters must be performed to fully account for uncertainty. Although this approach is rigorous, its high computational cost places it beyond the scope of the present study. Nonetheless, it remains a promising direction for future work in physics-informed kernel design and selection.

#### Mean Selection

The simplest information to include in the mean μ is the hard-particle-like repulsive shell and bond information. In simulations, bonds are often modeled using a harmonic oscillator, resulting in a sharp, approximately Gaussian peak in *g*(*r*). This leads to the bonded portion of the mean being represented as a sum of Normal distributions,

$$\mu_{\mathrm{Bonded}}\left(r\right)=\overset{B}{\underset{b=1}{\sum }}h_{b}N\left(r|r_{b},s_{b}\right)$$ 31

Here, the sum is taken over each unique structural peak in the particular molecule. For instance, when studying the oxygen–hydrogen correlation of water, we would expect at least one peak to correspond to the oxygen–hydrogen bond. However, for larger molecules, the situation can become more complex. Consider the hydrogen–hydrogen correlations in benzene. Although each hydrogen atom is exclusively bonded to carbon, we still observe bond-like peaks in *g*(*r*) due to the intramolecular hydrogen atoms still being in proximity with one another. These manifest as approximately normal peaks as if they were directly bonded. In principle, one could then relate the parameter *r*_*b*_ to the equilibrium bond lengths, *s*_*b*_ to the strength of the bonds, and *h*_*b*_ to the typical number of atoms at the distance *r*_*b*_. The excluded volume part is then represented as a simple sigmoid. This choice aligns with the limit behavior of the mean outlined above,

$$\mu_{\mathrm{Non‐Bonded}}\left(r\right)=\frac{1}{1+\exp\left(-s_{0}\left(r-r_{0}\right)\right)}$$ 32

Overall, the mean function for the GP model is then,

$$\mu \left(r\right)=\mu_{\mathrm{Bonded}}\left(r\right)+\mu_{\mathrm{Non‐Bonded}}\left(r\right)$$ 33

$$=\overset{B}{\underset{b=1}{\sum }}h_{b}N\left(r|r_{b},s_{b}\right)+\frac{1}{1+\exp\left(-s_{0}\left(r-r_{0}\right)\right)}$$ 34

Consequences of selecting this particular real-space GP mean on the structure factor are further discussed in Supporting Information Section S4.

#### Hyperparameter Optimization

The final step of the method involves inferring the GP hyperparameters, given the experimental structure factor. For this task, we learn a Bayesian hyperposterior using a hierarchical inference scheme (type II maximum likelihood) with appropriately defined priors as described in Supporting Information Section S3. The computational cost of the nonstationary GP method is dominated by this hyperparameter optimization step, which entails repeated cubic-scaling GP evaluations in the number of experimental *q*-space points. The difficulty of optimization grows with the number of hyperparameters, as this typically goes hand-in-hand with a rougher objective function. The numerical Fourier transform unique to this work scales linearly with the number of *r*-space grid points and is negligible in comparison to the matrix inverse.

Despite the theoretical complexity, in our experiments, we found the optimization to run end-to-end within a couple of hours on a laptop given modest-sized data sets (200–500 observations). However, for a given set of hyperparameters, a single GP inference is of relatively trivial computational cost given standard memory and processor capabilities of modern personal computers up to approximately 10^3^ observations. The establishment of standardized ranges or additional physical constraints on the hyperparameters would support rapid characterization of numerous samples with the added benefit of uncertainty quantification. Our implementation of the optimization and inference procedure is available on GitHub at https://github.com/hoepfnergroup/LiquidStructureGP-Sullivan.

## Results

Having established the theoretical framework, we now demonstrate its utility on both simple and complex liquids through synthetic and experimental scattering data. (1) In a liquid argon scattering experiment, we demonstrate excellent agreement between GP-derived structure factors and results from a gold-standard neutron scattering analysis. Beyond numerical accuracy, the nonstationary GP provides enhanced physical interpretability through kernel heat maps and posterior covariance matrices, which visualize the relationship between momentum- and real-space features. (2) To validate the nonstationary GP framework in a molecular system, we applied the method to simulated liquid water with a known ground truth. We find that the GP reconstructs the real-space RDF, even under moderate noise. (3) Finally, we apply the framework to an experimental X-ray scattering experiment of liquid water. Here, the GP yields a novel prediction for the oxygen–oxygen RDF with uncertainty quantification. The posterior is then propagated to estimate posterior predictive statistics for the first and second peak positions and heights, as well as the coordination number. Both the visualization of error bars using a noise-free posterior and the GP-based inference of coordination numbers are detailed in Supporting Information Sections S5 and S6, respectively.

### Liquid Argon

We begin by examining the quintessential neutron-weighted argon structure factor measured by Yarnell.^29^ Although the data set incorporates post hoc modifications to address multiple scattering, background scattering, finite sample volumes, and noise, it remains widely regarded as a benchmark data set in the field. However, a key issue is that denoising alters the uncertainty estimation in the nonstationary GP method. To approximate the original, predenoised data and preserve realistic uncertainty estimates, we reintroduced constant Gaussian noise (σ_noise_^2^ = 0.04) to the input estimated from a figure in Yarnell’s manuscript.

Now, for the nonstationary GP construction of liquid argon, which has no bonded contributions, the hyperparameter vector is reduced to $\theta ={\left[r_{0},s_{0},l,\mathrm{Max},\mathrm{Slope},\mathrm{Loc},\mathrm{Decay},\omega \right]}^{T}$ . Figure 2 visualizes eqs 18–21 before hyperparameter optimization. Notably, the kernel matrices exhibit the distinct structural characteristics enforced through the prior. Specifically, the lack of structure at low radius values in eqs 18 and 19 corresponds to the excluded volume of the argon atoms. The presence of this feature in the prior distribution suggests that the atomic size is learned during the LMLH optimization of the prior mean hyperparameters rather than conditioning on the observed data. At medium to large values of *r,* there is a clear periodic structure in *q*, indicating both positive and negative correlations. This is an expected feature due to the underlying integration factor in eq 1 being a decaying sinusoid. Lastly, notice the magnitudes involved in each correlation. While the maximum value in the *K*(*r*, *r′*) correlation is typical of Ar, the magnitudes in *K*(*q*, *q′*) are greater than what is observed in experiments. This results in increased flexibility in the low *q* region that is inconsistent with known limiting behaviors of *S*(*q*).

**Figure 2:**
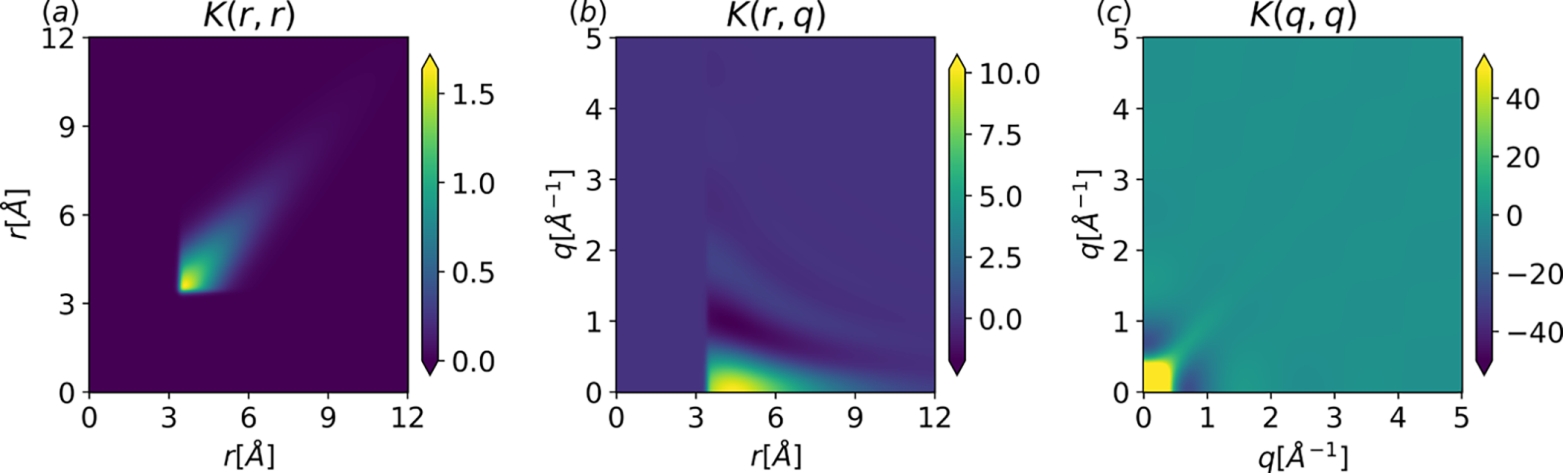
Gaussian process kernels after hyperparameter fitting of argon at a temperature *T* = 85 [K] and density ρ = 0.02125 [atom/Å^3^]. Left corresponds to eq 18, middle corresponds to eq 19, and right corresponds to eq 21. The color bar represents the range of values indicated by the colormap. Any values outside the specified range are clipped and displayed by using the colors corresponding to the nearest boundary.

For example, the high variance at low *q* (σ_*q*_^2^ > 40, Figure 2c) arises from model misspecification. This discrepancy appears to result from the absence of constraints on the total density and isothermal compressibility. To understand this, consider the case *q* = 0, where the sinc term in eq 1 tends to 1 in the *q* → 0 limit so that *S*(0) = 1 + 4πρ∫_0_^∞^(*g*(*r*) – 1)*r* ^2^d*r*. The behavior at *q* = 0 is then determined by the well-known compressibility equation, which relates *S*(*q*) to the isothermal compressibility, lim_*q*→0_ *S*(*q*) = ρ*k*_B_ *T*χ_*T*_. Hence, the large prior variance at *q* = 0 indicates that the function distribution does not have a fixed isothermal compressibility. Thermodynamic quantities of this type could be incorporated into the model directly as constraints, ensuring that all realizations of the experimental data are consistent with known thermodynamic quantities. Several strategies exist to enforce such consistency, including the use of warping functions, the design of kernel and mean functions that inherently satisfy the constraints, or the incorporation of auxiliary data to implicitly embed them.^41^ Although such constraints are not utilized here, they remain a promising direction for future exploration of GPs as mathematical models in liquid state theory.

With these kernels, we perform hyperparameter optimization by minimizing eq 10 (see Supporting Information Section S7 for a table of initial and trained hyperparameters), after which the prior distribution over structure factors is conditioned on the data to yield a posterior distribution, along with the associated distribution over the RDF. This procedure is described by eqs 11–13 and 22–24. The posterior mean and covariance in ***q*** and ***r*** space are presented in Figure 3.

**Figure 3:**
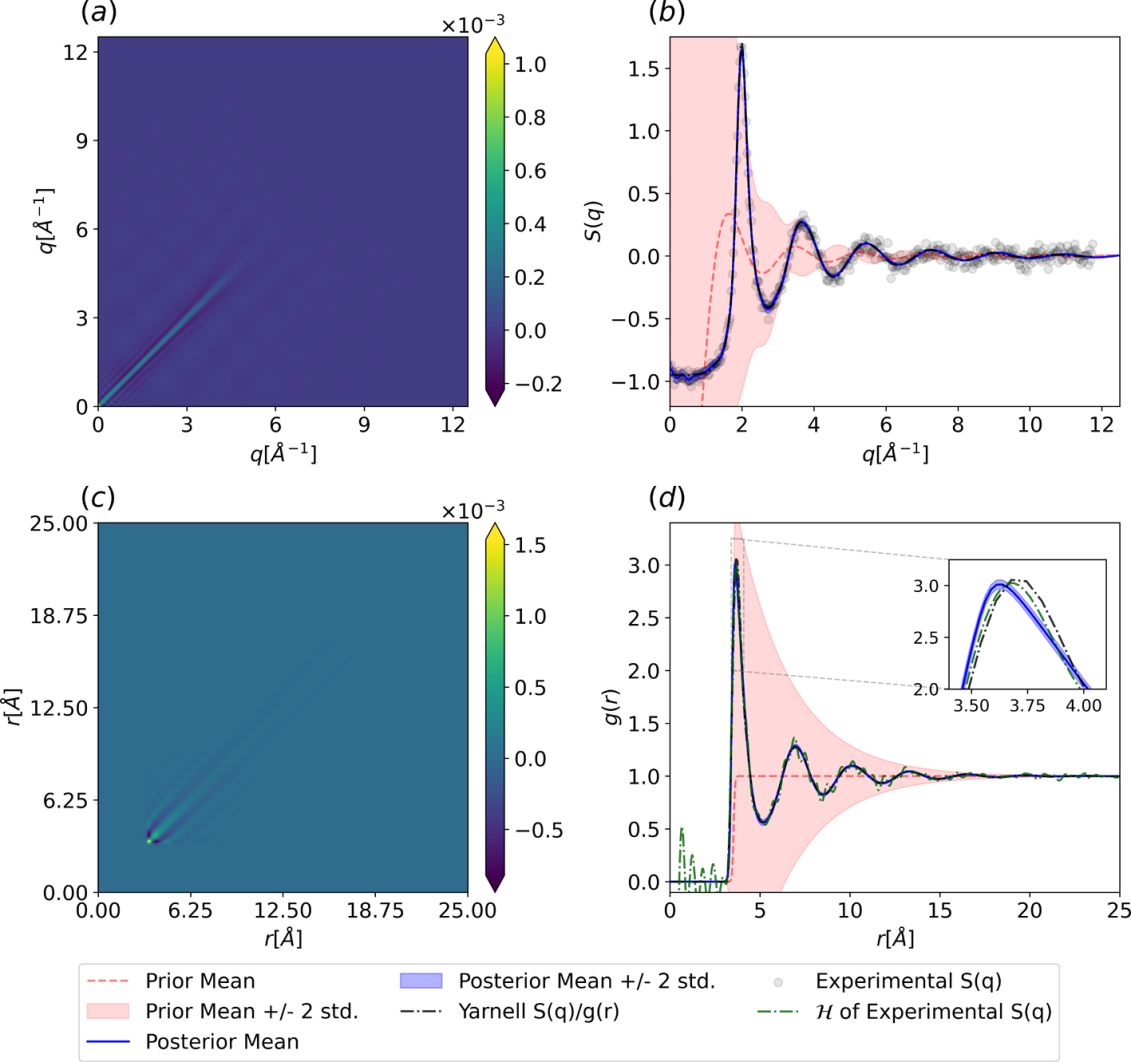
Posterior of the Gaussian process fit to argon at a temperature of *T* = 85 [K] and a density of ρ = 0.02125 [atom/Å^3^]. (a) Posterior covariance in *q*-space from eq 13. (b) Prior and posterior distributions for the argon structure factor from eq 12. (c) Posterior covariance in *r*-space from eq 24. (d) Prior and posterior argon RDF from eq 23. The color bars are clipped similarly to Figure 2.

The direct rFT of the data in Figure 3d clearly exhibits *q*_max_ cutoff errors, manifested as high-frequency oscillations. As commented by Lorch in 1969, these high-frequency oscillations are often “erroneously identified as truncation ripples by other workers.”^19^ In the Yarnell interpretation, truncation ripples were removed through an iterative procedure. First, the structure factor *S*(*q*) was artificially extended to the compressibility limit (*q* = 0), then directly Fourier-transformed to produce an initial estimate of *g*(*r*). Next, *g*(*r*) was set to zero in the low-*r* region (0 ≤ *r* ≤ 0.8*d*, where *d* is an estimated atomic diameter) and inverse Fourier-transformed back to yield an updated *S*(*q*). This process was repeated iteratively, until it was no longer necessary to set *g*(*r*) to zero at low-*r*. This iterative procedure is well-established in neutron scattering analysis, and the nonstationary GP framework naturally preserves the spirit of this procedure. The process of optimizing eq 10 formally uses the same scheme. In practice, both Yarnell’s method and the nonstationary GP yield nearly identical predictions of the real-space structure, with deviations likely arising from the denoising procedure or imperfect hyperparameter optimization. To account for this type of uncertainty in the GP formalism, one would increase the hierarchy of the optimization and propagate *p*(**θ**|*Y*) into the *g*(*r*) distribution. Due to the associated computational cost as well as the negligible difference to Yarnell’s results, we did not explore this avenue; however, it provides a clear direction for future work.

The nonstationary GP methodology also provides us with direct access to the posterior covariance matrix in real space. We can see in Figure 3c that the posterior covariance in real-space exhibits a highly nonstationary structure that is fully consistent with the physical constraints imposed in the kernel design stage. Namely, the zero covariance at short-range exactly mimics a certain low-*r* limit constraint, while the decaying covariance at high-*r* reflects the decay of the RDF oscillations to unity. In momentum space, the decreasing posterior covariance of the structure factor as a function of *q* demonstrates that the physics-informed prior on the RDF naturally leads to a physically consistent estimate of *S*(*q*) through the rFT (Figure 3a).

### Water

We now turn our attention to liquid water, a considerably more complex system due to the presence of chemical bonds and three partial structure factors. When inferring real-space structures in bonded systems, the nonstationary GP framework requires an additional prior mean term, given by eq 31, to handle the bonded part of the structure for the oxygen–hydrogen and hydrogen–hydrogen partial structure factors. Other than the additional hyperparameters introduced by the prior mean, the nonstationary GP regression proceeds in the exact same manner as in the liquid argon case.

#### Simulated Liquid Water

First, we analyzed an artificial noisy unweighted structure of simulated water obtained from the flexible TIP4P/2005f water model^44^ (for simulation and GP training details, see Supporting Information Section S8). The goal of this test was to verify that the nonstationary GP accurately recovers the ground truth real-space structure from noisy momentum-space data. This validation step is essential, as it builds confidence in the methodology before applying it to experimental data where the ground truth real-space structure is unknown.

In Figure 4, we show the posterior covariance and hydrogen–hydrogen partial structure factor and RDF including the GP prior (red), posterior (blue), perturbed structure factor (black dots), and ground truth (dashed black line). The hydrogen–hydrogen partial structure factor is presented here because it includes both bonded and nonbonded contributions, resulting in a more complex correlation structure with more hyperparameters than the oxygen–oxygen case, and thus poses a greater challenge for the method. The data in Figure 4b clearly show that the hyperparameter learning and regression result in an excellent posterior representation of the hydrogen–hydrogen partial structure factor as well as its rFT to real space in Figure 4d. The bonded and nonbonded regions of the hydrogen–hydrogen partial RDF are well captured by the model, with the ground truth lying within the estimated RDF posterior distribution. While we only show the hydrogen–hydrogen partial posterior distributions here, we note that the oxygen–hydrogen and oxygen–oxygen posterior distributions exhibit strong qualitative agreement and are provided in Supporting Information Section S8.

**Figure 4:**
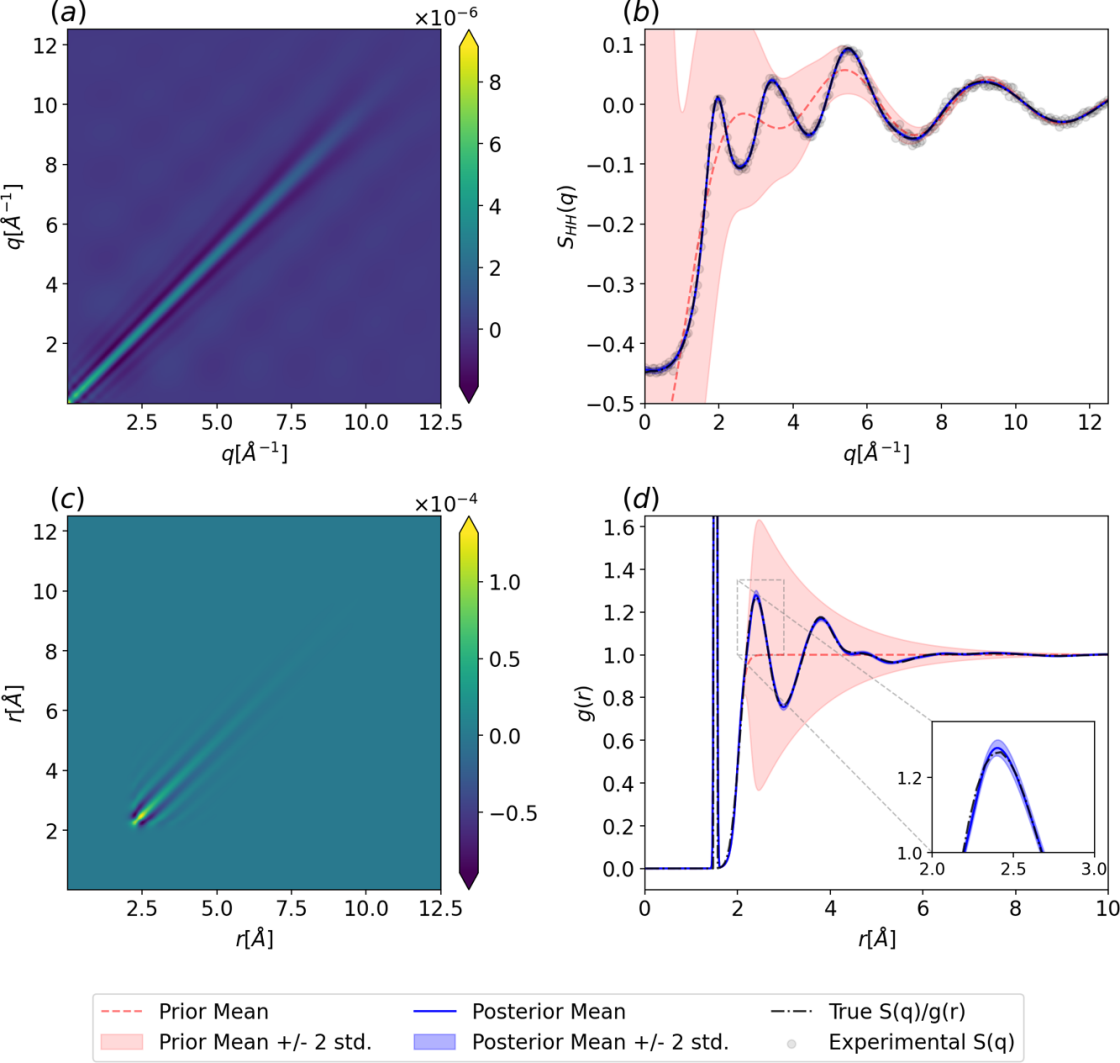
Posterior of the Gaussian process fit to structure factors derived from NVT simulations at a temperature of 298.15 K and density of 1 g cm^–3^ with flexible TIP4P/2005f water. (a) Posterior covariance in *q*-space from eq 13. (b) Prior and posterior distributions for the hydrogen–hydrogen structure factor from eq 12. (c) Posterior covariance in *r*-space from eq 24. (d) Prior and posterior hydrogen–hydrogen RDF from eq 23. The sharp feature observed at ∼1.6 Å represents the distance between the hydrogen atoms in a water molecule.

#### Experimental X-ray Scattering of Liquid Water

We now turn to the analysis of a broadened X-ray scattering data set for liquid water reported by Skinner and co-workers.^23^ In X-ray scattering experiments on water, the signal is dominated by oxygen–oxygen correlations due to the weak scattering cross section of hydrogen arising from its single electron. As a result, assuming that background scattering corrections in the original data set were appropriately handled, the nonstationary GP model in this case needs only to infer the oxygen–oxygen correlation. We then compared our predictions to those of Skinner’s interpretation. In their analysis, a variable Lorch modification function developed by Soper and Barney^21^ was applied with fixed parameters (*a* = 2.8 and *b* = 0.5 Å), and error propagation was performed using the method of Weitkamp.^25^

The nonstationary GP applied to the structure factor provides an excellent representation of the underlying structure given the noisy experimental X-ray scattering data (for GP training details, see Supporting Information Section S9). More insightful, however, is the comparison between the nonstationary GP model and Skinner’s interpretation shown in Figure 5d,e. While the mean predictions from both methods closely align at and beyond the first peak, significant differences emerge at lower distances. Specifically, Skinner’s interpretation exhibits nonphysical oscillations below the collision diameter of the oxygen atom, an artifact of Fourier truncation, that leads to nonphysical negative values of *g*(*r*). Even more striking are the differences in uncertainty estimates between the two methods. Skinner’s uncertainty rapidly increases at low-*r*, primarily reflecting known limitations of the applied error-estimation procedure,^25^ making it unclear whether these uncertainties genuinely represent data-informed variability or methodological artifacts. Conversely, the nonstationary GP uncertainty profile matches physical expectations: negligible uncertainty at low-*r*, a pronounced increase reaching a maximum around the first peak, followed by a gradual decay to negligible uncertainty at large distances. Notably, our interpretation predicts a slightly larger uncertainty in the local structure of water in the first solvation shell.

**Figure 5:**
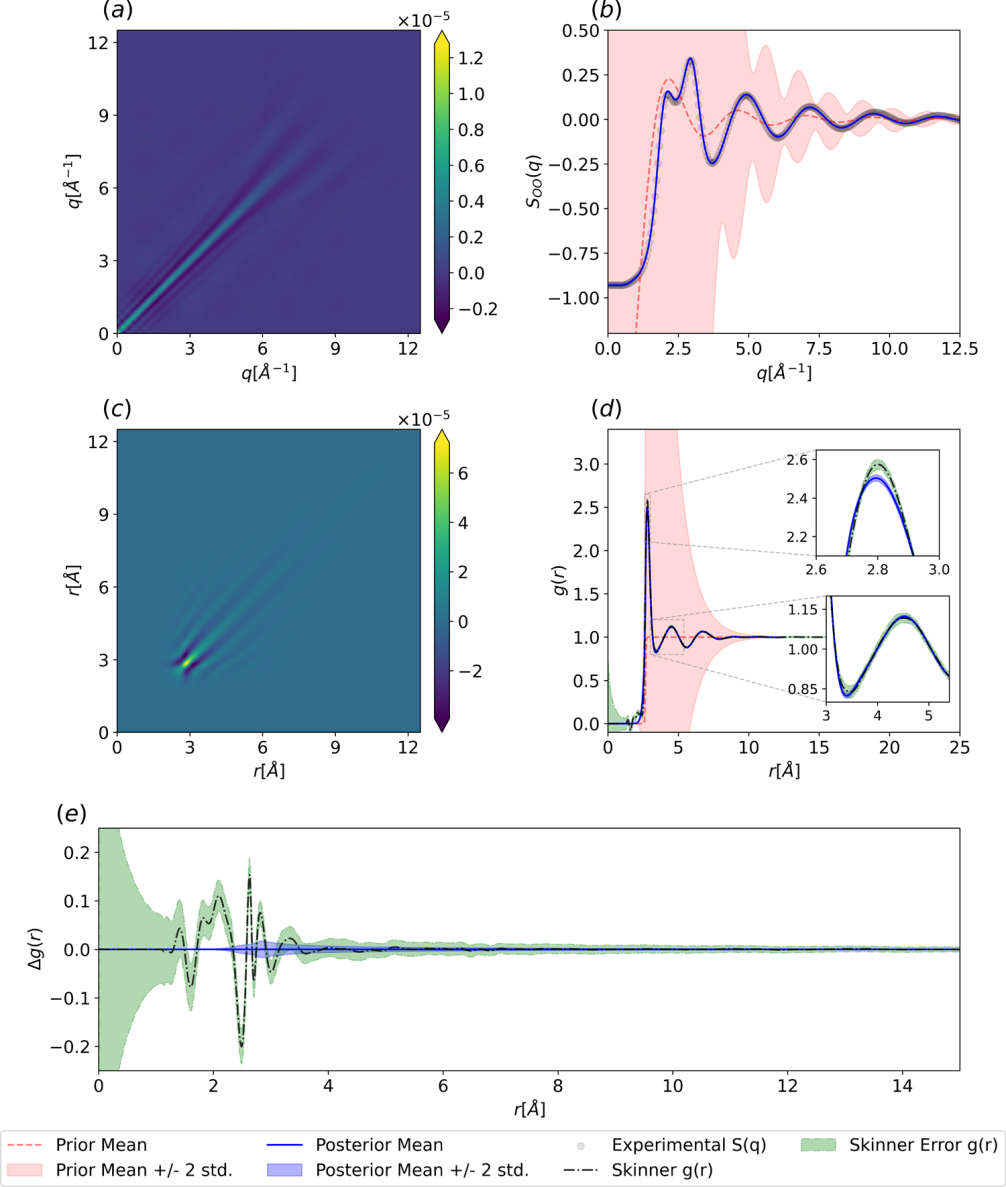
Posterior of the Gaussian process fit to the X-ray scattering data. (a) Posterior covariance in *q*-space from eq 13. (b) Prior and posterior distributions for the oxygen–oxygen structure factor from eq 12. (c) Posterior covariance in *r*-space from eq 24. (d) Prior and posterior oxygen–oxygen RDF from eq 23. (e) GP Mean subtracted comparison between the uncertainty estimates from the nonstationary GP approach and Skinner’s interpretation.^23^

A physically justified posterior distribution on the RDF can subsequently be used to estimate statistics on other observables, such as peak heights and peak positions (Figure 6) as well as coordination number (Figure 7). Since the distributions are nearly Gaussian, we present the mean plus or minus two standard deviations (μ ± 2σ) as a summary statistic. The first peak location is estimated to be 2.793 ± 0.002, and the first peak height is 2.505 ± 0.016. The joint 2D marginal distributions over peak location and peak height show near-zero correlation for every two parameter sets, aside from a slight positive correlation (0.27) between the first peak height and first peak location. Finally, the estimated first coordination number is 4.722 ± 0.07, which is in good agreement with the generally accepted value determined from X-ray scattering data of 4.7.^45^

**Figure 6:**
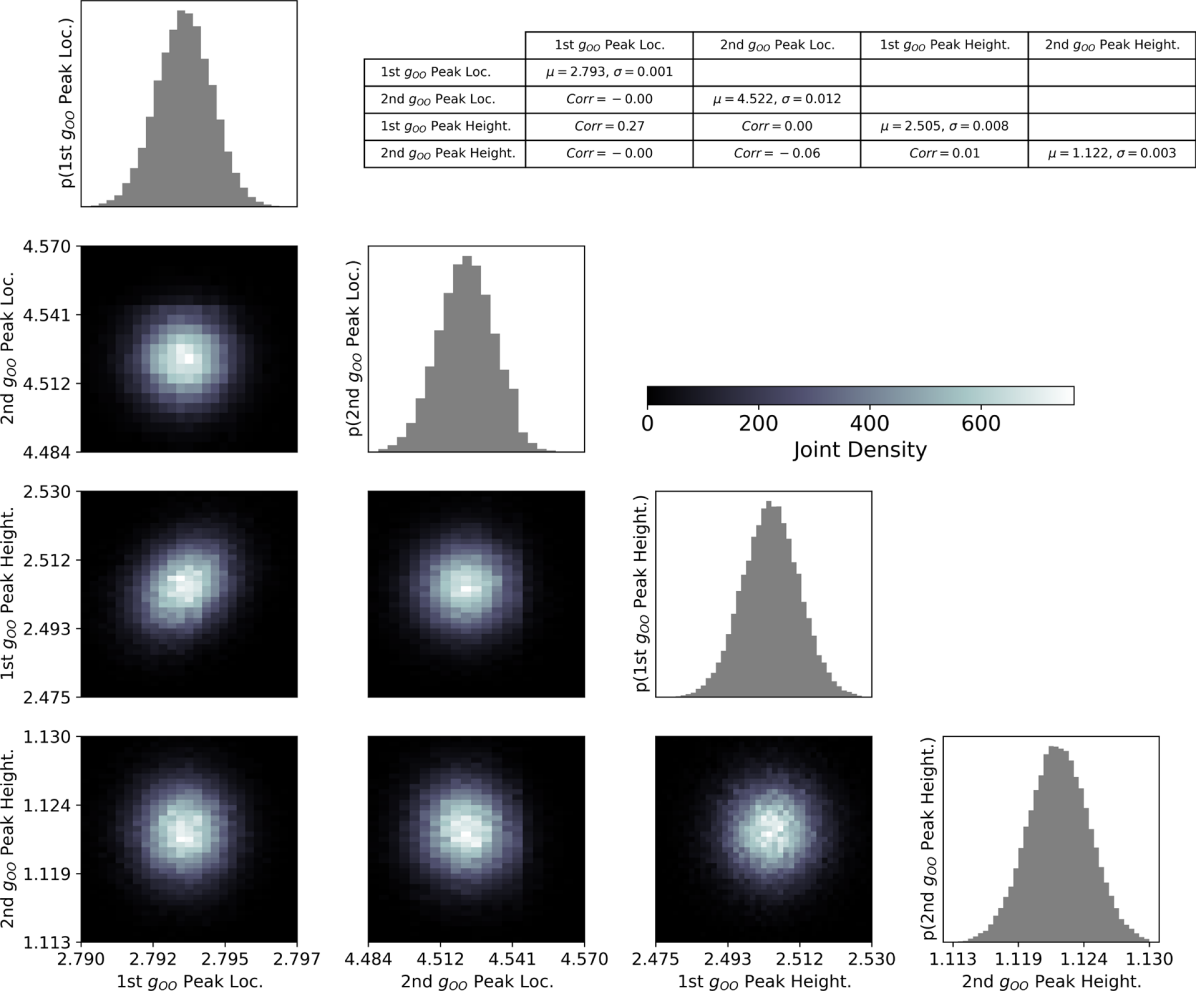
This corner plot shows the joint distribution of the first and second RDF peak locations and heights. The table shows the mean and standard deviation values of the marginals along its diagonal. The off-diagonal terms are the Pearson correlation coefficients in the joint marginal distributions.

**Figure 7:**
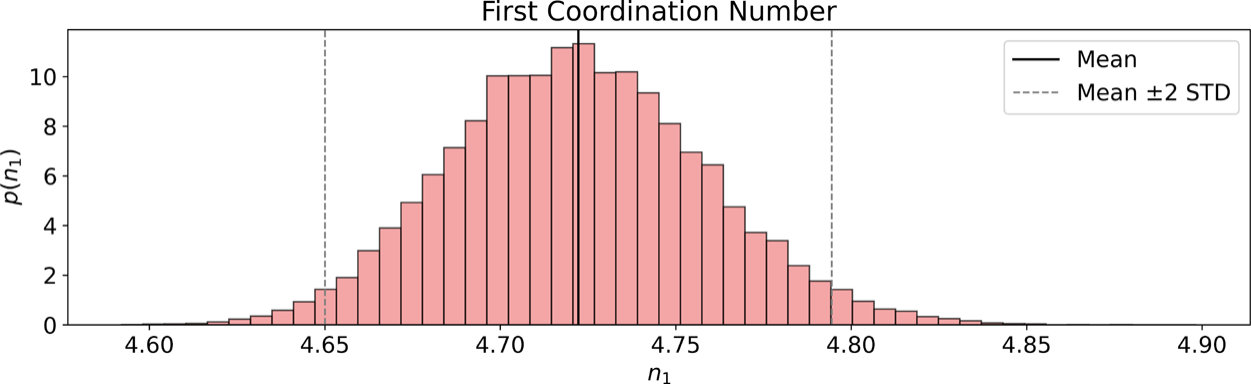
Histogram estimation of the coordination number probability density derived from samples of the nonstationary GP posterior.

Given that water is of central importance for a wide variety of fields and is the subject of substantial investigation over its local structure, it is worth reflecting on which structural interpretations should serve as benchmarks for molecular modeling. The nonstationary GP framework presented here offers not only a physics-informed reconstruction of the RDF, but also a posterior predictive distribution that quantifies uncertainty in a principled way. This makes it particularly well-suited for benchmarking simulations, where one expects model predictions -- in this case RDFs, coordination numbers, peak heights, and peak positions -- to fall within credible intervals that reflect both experimental noise and structural ambiguity. While no interpretation is free from assumptions or limitations, the GP-based approach provides a transparent and reproducible framework that balances physics with statistical rigor and is likely to be a more robust choice for comparison against molecular models.

## Discussion

The nonstationary GP framework offers a principled, data-driven approach to structural inference by combining Bayesian inference with physics-informed priors. We demonstrate accurate fits for test systems ranging from liquid Ar and TIP4P/2005f water to experimental X-ray scattering of liquid water with only modest physical assumptions. The method operates on a continuous domain in both momentum and real space to mitigate problems with binning artifacts or *q*_max_ truncation errors. The model effectively filters normal random noise present in the experimental observations, which ultimately stabilizes any subsequent analysis of downstream properties, such as peak heights, locations, and coordination numbers. By avoiding simulation-specific biases (e.g., from force field parameters, thermostats, or numerical integrators), the GP posterior provides a reproducible and physically grounded benchmark. Moreover, its flexibility allows for the inclusion of additional physics-based constraints (e.g., isothermal compressibility limits, virial equations, Kirkwood–Buff integrals^4^), making it a powerful tool for bridging scattering data with macroscopic thermodynamic properties.

The nonstationary GP method also hints at a deeper connection between structure and interatomic potentials when there is noise present in the data. The Henderson inverse theorem, which shows that the RDF for pairwise additive and homogeneous systems has a pair potential that is unique up to an additive constant, is derived in the noiseless limit.^2^ However, as shown empirically by Soper, when noise is present, there is an ensemble of potential energy functions corresponding to the scattering data target.^46^ We hypothesize that the nonstationary GP method can be considered a Laplace approximation on the true structural posterior determined by such a potential energy ensemble. Adopting this philosophical perspective could significantly enhance our understanding of structure–thermodynamics relationships and improve the accuracy of thermodynamic predictions.

Despite these advantages, several limitations remain. The nature of scattering experiments themselves poses the problem that species with low scattering length densities may be effectively invisible in the total signal. Additionally, all structure-analysis methods confront the underdetermined nature of the Faber–Ziman decomposition for multicomponent systems and mixtures, which permits multiple RDF solutions consistent with the same total scattering data, leading to nonuniqueness.^47^ The GP framework could be extended to these cases by representing each partial structure factor as a linear combination of nonstationary GPs; however, this approach quickly increases the number of hyperparameters to be inferred, leading to higher computational cost. It may also be possible to develop methods analogous to EPSR that consistently integrate experimental data with molecular models of the interatomic potential within a Bayesian framework (c.f. ref 32), though, to our knowledge, no such algorithm has been attempted.

Furthermore, absent *perfect* experimental data processing procedures (e.g., background, multiple scattering, inelasticity, etc.) and scattering statistics resulting from an infinite radiation flux, no fluid structure interpretation can be entirely unbiased. While our approach assumes that these corrections are accurate, extending the GP framework to model them explicitly would be a valuable step toward a more complete and uncertainty-aware analysis. In principle, the GP framework can incorporate such systematic corrections hierarchically, for example, by using non-Gaussian likelihoods for scattering corrections, performing Bayesian inference over parametric models of systematic errors, and estimating time-of-flight uncertainty via error-in-variables approaches.

Finally, the GP prior mean and kernel used here represent just one of many choices that can satisfy the physical constraints. Future work could explore alternative priors that more tightly enforce thermodynamic behavior or integrate knowledge of interatomic potentials obtained through other Bayesian schemes.^26,32^ As the methodology evolves, refining and exploring alternative priors are likely to improve interpretability, computational efficiency, and predictive accuracy.

## Conclusions

We introduce a method for rigorous uncertainty quantification and propagation in experimentally derived radial distribution functions using physics-informed, nonstationary GP regression. This approach constructs a minimal yet physically expressive kernel that preserves the Fourier duality between the structure factor and the RDF. By addressing pervasive challenges in the Fourier transformation of momentum-space scattering data and incorporating Bayesian inference, our approach offers a robust and interpretable alternative to traditional structural analysis methods.

Applied to both simple and complex liquids, the model yields physically reasonable posterior distributions for radial distribution functions that capture both mean behavior and structural uncertainty. Crucially, the nonstationary GP framework achieves this without relying on computationally intensive molecular simulations that may be affected by systematic model bias imposed by force field assumptions. Its flexibility allows for the principled incorporation of physical knowledge and integration of data preprocessing steps through hierarchical modeling. Taken together, we conclude that the Bayesian framework established in this study may represent the best path toward an unbiased assessment of fluid structure.

## Supplementary Material





## Data Availability

Structure factors, radial distribution functions with credibility intervals, posterior means and covariances, and nonstationary GP hyperparameters are available in the Supporting Information, from the corresponding authors upon reasonable request, and also provided on Github at https://github.com/hoepfnergroup/LiquidStructureGP-Sullivan.

## Footnotes

1 For a concise list of notation used in this manuscript, see Supporting Information Section S1.

2 Notably one can extend the domain of the FT to the tempered distributions, we do not consider such cases in this work.

## Notes

Python notebook for importing.txt files of the posterior mean and covariances for liquid argon, TIP4P–F water, and liquid water at atmospheric pressure and 295 K (ZIP)
Section S1: notation; Section S2: deriving the radial Fourier transform operator; Section S3: numerical considerations and hyperparameter optimization; Section S4: discussion on prior mean selection; Section S5: understanding error bar visualization for GPs; Section S6: GP coordination number analysis; Section S7: neutron weighted argon structure analysis (Table S1: initial and final GP hyperparameters; Figure S1: optimization analysis, posteriors, and residuals); Section S8: simulated unweighted water data set (Figure S2: simulated oxygen–oxygen predictions; Figure S3: simulated oxygen–hydrogen predictions; Figures S4–S6: optimization progression, posteriors and residuals for the oxygen–oxygen, oxygen–hydrogen, and hydrogen–hydrogen partials, respectively); Section S9 debroadened X-ray water analysis (Table S2: initial and final GP hyperparameters; Figure S7: optimization analysis, posteriors, and residuals) (PDF)
