# Supplementary material for: Physics-Informed Gaussian Process Inference of Liquid Structure from Scattering Data
Source: J Phys Chem B. 2025 Oct 31;129(45):11802–15. doi: 10.1021/acs.jpcb.5c05024 (PMC12621243; doi:10.1021/acs.jpcb.5c05024)
Supplement: Supplementary file 2 [file jp5c05024_si_002.pdf]

Supporting Information: Physics-Informed Gaussian Process Inference of Liquid Structure from Scattering Data

Harry Winston Sullivan,[†] Matej Cervenka,[‡] Brennon L. Shanks,[*,‡] and Michael P. Hoepfner[*,¶]

†*Department of Chemical Engineering and Material Science, University of Minnesota - Twin Cities, Minneapolis, MN*

‡*Institute of Organic Chemistry and Biochemistry of the Czech Academy of Sciences, Flemingovo nám. 2, 166 10 Prague 6, Czech Republic*

¶*Department of Chemical Engineering, University of Utah, Salt Lake City, UT*

E-mail: shanks.brennon@uochb.cas.cz; hoepfner@chemeng.utah.edu

S1. Notation

- A symbol f is a scalar.
- A bold symbol $\boldsymbol{f}$ is a column vector.
- A bold symbol with a hat $\hat{\boldsymbol{f}}$ is a matrix.
- A bold symbol with a tilde $\tilde{\boldsymbol{f}}$ is an operator that acts on functions. The arguments of an operator are put between [ ], arguments to the right of a semicolon ; indicate implicit arguments of the operator. Implicit arguments are not exhaustive and are purely shown for pedagogy.
- An operator with a subscript $\tilde{\boldsymbol{f}}_x$ is meant to indicate that it is treating the argument as a function of the subscript alone. For example $\tilde{\boldsymbol{f}}_x[g(x, y, z)]$ is the result of acting with $\tilde{\boldsymbol{f}}$ on the function $x \mapsto g(x, y, z)$ which has implicit dependence on y and z.
- A numerical approximation to an operator is indicated with a blackboard bold $\mho$. While the notation makes it look as if it acts on functions it is implied that it acts on discretized evaluations of the them.
- The abuse of notation $f(\boldsymbol{x})$ just means $[f(x_1), \ldots, f(x_n)]^T$.
- $\boldsymbol{f} \sim p(\boldsymbol{f} \mid \boldsymbol{\zeta}_1, \boldsymbol{\zeta}_2, \ldots)$ means the column vector $\boldsymbol{f}$ is distributed by the p.d.f. p parametrized by $\boldsymbol{\zeta}_1, \boldsymbol{\zeta}_2, \ldots$. This can be interpreted in a Bayesian manner by considering the random variable $\boldsymbol{f}$ to be conditioned on by the set of $\boldsymbol{\zeta}$s.
- $\operatorname{cov}(f(x), g(y))$ is the covariance between the function values $f(x)$ and $g(y)$.
- $f \sim \mathcal{GP}(\mu, K)$ means the function $f(x)$ is a Gaussian process whose finite index set $\boldsymbol{x}$ has function values distributed as a multivariate Gaussian with mean $\mu(\boldsymbol{x})$ and covariance $K(\boldsymbol{x}, \boldsymbol{x})$.

- $\mathbb{R}^d$ is the whole real line in d dimensions. If there is no superscript then it is one dimensional.
- $\mathbb{R}^{d,+}$ is the non-negative real line in d dimensions. If there is no d in the superscript then it is one dimensional.
- An integral without bounds, such as $\int p(\boldsymbol{x})d\boldsymbol{x}$, implies it is taken over the whole support of the distribution.

S2. Derivation of the rFT Operator

Each of the partial structure factors $S_{\alpha,\beta}(\boldsymbol{q})$is related to the RDF $g_{\alpha,\beta}(\boldsymbol{r})$ via a standard 3D inverse Fourier transform.

$$S_{\alpha,\beta}(\boldsymbol{q}) - 1 = \rho \int (g_{\alpha,\beta}(\boldsymbol{r}) - 1) \exp(i\boldsymbol{q} \cdot \boldsymbol{r}) d\boldsymbol{r} \tag{1}$$

If the material is isotropic then we may write this in terms of $r = |\boldsymbol{r}|$ and $q = |\boldsymbol{q}|$. To do this first consider a spherical polar co-ordinate system in real space where the polar angle points in the direction of $\boldsymbol{q}$. We can choose θ to be the angle between these two vectors.

$$= 2\pi\rho \int_{r=0}^{\infty} \int_{\theta=0}^{\pi} (g_{\alpha,\beta}(r) - 1) \exp(iqr\cos(\theta)) r^2 \sin(\theta) dr d\theta \tag{2}$$

Performing the integral over theta will give

$$S_{\alpha,\beta}(q) - 1 = 4\pi\rho \int_{r=0}^{\infty} (g_{\alpha,\beta}(r) - 1) \frac{\sin(qr)}{qr} r^2 dr \tag{3}$$

This implies the 3D Fourier transform of the $S(q)$ will give the $g(r)$

$$g_{\alpha,\beta}(r) - 1 = \frac{1}{2\pi^2\rho} \int_{q=0}^{\infty} (S_{\alpha,\beta}(q) - 1) \frac{\sin(qr)}{qr} q^2 dq \tag{4}$$

The difference in prefactor is due to the forward transform carrying the $\frac{1}{(2\pi)^3}$. This can be written in terms of the radial Fourier transform (rFT) operator

$$\tilde{\mathcal{H}}_q[f(q)] = \frac{1}{2\pi^2\rho}\int_0^\infty f(q)\frac{\sin(qr)}{qr}q^2dq, \quad \tilde{\mathcal{H}}_r^{-1}[f(r)] = 4\pi\rho\int_0^\infty f(r)\frac{\sin(qr)}{qr}r^2dr. \tag{5}$$

The RDF structure factor relationship is then written succinctly as

$$S_{\alpha,\beta}(q) = 1 + \tilde{\mathcal{H}}_r^{-1}[g_{\alpha,\beta}(r) - 1], \quad g_{\alpha,\beta}(r) = 1 + \tilde{\mathcal{H}}_q[S_{\alpha,\beta}(q) - 1]. \tag{6}$$

S3. Mitigation of Numerical Errors and Hyperparameter Optimization

Although the GP formalism is well founded, it is still subject to the same constraints as any high-dimensional linear algebra program. The key issues we are concerned with include (but are not limited to) floating point errors, approximate sparsity of the kernel, unbounded derivatives, near-positive definiteness of matrices, and convergence of eigenvalues and eigenvectors. Often these aspects are left behind the curtain, only for the users to discover and solve on their own. Rather than following a *do-it-yourself* approach, we are including these details to avoid potential issues in future implementations of the GP paradigm.

Kernels

Kernel functions, and their corresponding kernel matrices, must be positive semi-definite (PSD) to ensure that they represent valid covariance matrices. Suppose we had a kernel function that is not PSD. This would imply the variance in the direction of at least one eigenvector would be negative, which is clearly nonsense probabilistically speaking. The approximate sparsity of our kernel, with many elements close to zero, may lead to a non-PSD matrix. To address this, we iteratively adjust the eigenvalues of non-PSD kernels. To do this

first compute the eigenvectors $\boldsymbol{v}_i$ and eigenvalues λ_i of the symmetrized kernel matrix.

$$\text{eigh}\left(\frac{\hat{\boldsymbol{K}} + \hat{\boldsymbol{K}}^T}{2}\right) = \{\lambda_i, \boldsymbol{v}_i\} \tag{7}$$

Then, we adjust each eigenvalue by adding the negative of the minimum eigenvalue plus a small offset, ε.

$$\lambda_{i,\text{Reconstruction}} = \lambda_i - \min(\lambda_i) + \varepsilon \tag{8}$$

We can then reconstruct the matrix from the adjusted eigenvalues and eigenvectors. If the result is still not PSD, we repeat the reconstruction with a slightly larger ε. Effectively this is a post-hoc shrinkage method for covariance estimation. This adjustment introduces additive, uncorrelated, normally distributed noise in the GP samples.

However, there is no free lunch. Care must be taken during this step; if the underlying kernel function (and its associated hyperparameters) pathologically produces nearly singular or numerically non-PSD matrices, the ε jitter term may grow large. This becomes increasingly relevant if the jitter term approaches the magnitude of the experimental noise ω, which can compromise the reliability of the uncertainty quantification. Additionally, computing the log marginal likelihood now depends heavily on the eigenvalue decomposition of the kernel matrix. This reliance introduces sensitivity to degenerate eigenvalues, which can cause unbounded derivatives with respect to the hyperparameters. Such unbounded behavior leads to instability in the optimization process, where large or erratic gradients may cause oscillations or divergence. We opt to mitigate these derivative issues by bypassing the gradient with respect to the post-hoc shrinkage all together. By overwriting the automatic differentiation with the identity we can prevent any unboundedness arising from degeneracy. Unboundedness can also result from repeatedly applying exponential functions in both the mean and kernel. To address this, it's useful to apply clamping functions to the inputs before taking the exponential, which prevents NaNs and infinite derivatives from disrupting

optimization without significantly affecting the typical output.

Hyperparameter Optimization

Machine learning inevitably comes down to inferring a robust set of hyperparameters conditioned on available observations, and non-stationary GP regression is no exception. In a fully Bayesian formalism, one can infer hyperparameter posterior distributions and propagate their uncertainty to the GP model via hierarchical Bayesian inference. The first step is a Bayesian inversion of the model evidence, $p(\boldsymbol{Y}|\boldsymbol{\theta})$, to give the conditional probability of the hyperparameters given available data, $p(\boldsymbol{\theta}|\boldsymbol{Y})$. Conceptually, we are asking *what is the probability that the hyperparameters in our prior model generated the training data?* Using Bayes' theorem, we can write the hyperposterior probability in log space as,

$$\log p(\boldsymbol{\theta}|\boldsymbol{Y}) = -\log(Z) - \underbrace{\frac{1}{2}(\boldsymbol{Y}-\boldsymbol{\mu}_{\boldsymbol{q}})^T(\hat{\boldsymbol{K}}_{\boldsymbol{q},\boldsymbol{q}}+\omega^2\hat{\boldsymbol{I}})^{-1}(\boldsymbol{Y}-\boldsymbol{\mu}_{\boldsymbol{q}})}_{\textit{Data Fit}} - \underbrace{\frac{1}{2}\log\det|\hat{\boldsymbol{K}}_{\boldsymbol{q},\boldsymbol{q}}|}_{\textit{Complexity Penalty}} - \frac{d}{2}\log 2\pi + \underbrace{\log p(\boldsymbol{\theta})}_{\textit{Prior Belief}} \tag{9}$$

where Z is a normalization constant. By inspection, it is clear that this expression represents a compromise between a data fit and a complexity penalty. As the model improves at matching the training data the *Data Fit* term decreases, overall increasing the log probability. Simultaneously, as the kernel becomes more complex, the determinant in the *Complexity Penalty* will increase, in turn decreasing the log probability. The determinant increase is associated with a larger volume spanned by the eigenvectors of the kernel matrix. The overall expression becomes a competition between these two terms, resulting in an Occam's razor effect that pushes the model towards the simplest explanation of the dataset.

Unfortunately, exact computation of the log probability is not tractable for generic choices of $p(\boldsymbol{\theta})$, $\hat{\boldsymbol{K}}_{\boldsymbol{q},\boldsymbol{q}}$, and $\boldsymbol{\mu}_{\boldsymbol{q}}$. The root of the problem lies in the computation of the normalization constant Z via integration of $p(\boldsymbol{Y}|\boldsymbol{\theta})p(\boldsymbol{\theta})$ over $\boldsymbol{\theta}$, which becomes computationally infeasible even for relatively simple GPs. Therefore, a point estimate of $\boldsymbol{\theta}$ was inferred and used for

subsequent GP modeling by maximizing the log hyperposterior with gradient ascent applied to eq (9). This process is outlined in the primary loop shown in the process flow diagram of the main manuscript.

Utilization of a numerical rFT technique allows us to rewrite the optimization target stemming from eq (9) as,

$$\begin{aligned}\arg\max_{\boldsymbol{\theta}} \Bigg[\frac{1}{2}\left(\boldsymbol{Y} - \tilde{\mathbb{H}}_r^{-1}[\boldsymbol{\mu}_{\boldsymbol{r}}]\right)^T \left(\tilde{\mathbb{H}}_r^{-1}\left[\tilde{\mathbb{H}}_r^{-1}[\hat{\boldsymbol{K}}_{\boldsymbol{r},\boldsymbol{r}}]\right] + \omega^2\hat{\boldsymbol{I}}\right)^{-1}\left(\boldsymbol{Y} - \tilde{\mathbb{H}}_r^{-1}[\boldsymbol{\mu}_{\boldsymbol{r}}]\right) \\ - \frac{1}{2}\log\det\left|\left(\tilde{\mathbb{H}}_r^{-1}\left[\tilde{\mathbb{H}}_r^{-1}[\hat{\boldsymbol{K}}_{\boldsymbol{r},\boldsymbol{r}}]\right] + \omega^2\hat{\boldsymbol{I}}\right)^{-1}\right| + \log p(\boldsymbol{\theta})\Bigg]\end{aligned} \tag{10}$$

where the hyperparameter vector is,

$$\boldsymbol{\theta} = [r_0, s_0, h_1, r_1, s_1, h_2, r_2, s_2, \ldots h_B, r_B, s_B, \ell, \text{Max}, \text{Slope}, \text{Loc}, \text{Decay}, \omega]^T. \tag{11}$$

The inclusion of ω is meant to treat the standard deviation in the likelihood as a learnable hyperparameter.

By employing automatic differentiation (AD), we can compute the gradients of the optimization target with respect to the hyperparameters in a precise and computationally efficient manner. This capability allows us to leverage gradient-based optimization algorithms, such as stochastic gradient descent or Adam,[1] to update the hyperparameters iteratively. The primary advantage of using AD is its ability to provide accurate gradients without the need for numerical differentiation, which can be sensitive to perturbations and often requires additional function evaluations. With AD, gradient computation is integrated into the optimization routine, enabling more rapid convergence to optimal hyperparameter values. Furthermore, AD facilitates the inclusion of our custom kernel, enhancing the flexibility of our modeling approach.

One challenge with hyperparameter optimization using this method is the risk of getting stuck in local minima, making a good initial guess crucial to training an accurate model. In

machine learning models like neural networks with many, non-interpretable hyperparameters, this is difficult task. However, since the GP mean and kernel functions are based on physical properties, chemical intuition makes finding an initial estimate straightforward. For example, a RDF computed from a single molecular dynamics simulation of the target system can be used to provide a good first guess for the kernel hyperparameters that can be subsequently refined according to the experimental scattering data. By first optimizing $\boldsymbol{\theta}$ in real space using simulation data you avoid the need for a grid choice as well as any discrete Fourier transform procedure. Heuristically we expect this to get $\boldsymbol{\theta}$ near it maximum-a-priori location, reducing the amount of optimization steps required to be taken on an experimental dataset. This physical interpretability greatly simplifies the hyperparameter training process in the non-stationary GP framework.

With these subtleties addressed, we also encounter the issue of non-physical hyperparameters arising from the optimization process. Traditionally, this is managed by setting the prior to assign zero probability to non-physical values, thereby avoiding their selection. However, this approach conflicts with the typical structure of stochastic optimizers, which operate over parameter vectors in $\mathbb{R}^d$. To work around this, we redefine each hyperparameter in terms of an unbounded "raw" parameter, optimizing in the raw space instead. Specifically, we apply a scaled sigmoid transformation and its logit inverse for each hyperparameter, such that forward and inverse transformations are given by

$$\boldsymbol{\theta}_i = \frac{u_i - l_i}{1 + \exp(-\boldsymbol{\theta}_{i,\mathrm{Raw}})} + l_i, \qquad \boldsymbol{\theta}_{i,\mathrm{Raw}} = \ln \frac{\frac{\boldsymbol{\theta}_i - l_i}{u_i - l_i}}{1 - \frac{\boldsymbol{\theta}_i - l_i}{u_i - l_i}} \tag{12}$$

where u_i and l_i are the upper and lower bounds, and $\boldsymbol{\theta}_{i,\mathrm{Raw}}$ is the unbounded parameter optimized with automatic differentiation. The last point we wish to make in this section is to note the requirement of Cholesky decomposition. One should never use a standard inversion algorithm on a kernel function. Paraphrasing Philipp Hennig, the use of a standard matrix inversion when computing a GP posterior should be considered a bug. The numerical

instability introduced by such a procedure is immense and should be avoided. The positive definiteness of kernel matrices allows us the luxury of a Cholesky decomposition for the computation of the posterior instead. In all transparency, the application of the eigenvalue decomposition above may introduce similar errors to the standard matrix inversion we are avoiding with Cholesky decomposition.

In practice, we have found that deviations from the ground truth often occur in regions near the first RDF peak or the transition from bonded to non-bonded structures. This effect is due to insufficient hyperparameter tuning, such as improper learning rates or early stopping. Not learning these sharp uptick regions precisely may seem unimportant when the quality-of-fit for the majority of the RDF is excellent, however; our prior work has demonstrated that this region can inform predictions of the repulsive exponent in a (λ-6) Mie potential.[2] This region is also important for liquid water, where nuclear quantum effects (NQEs) involving the light hydrogen atoms have been shown to significantly broaden structural features in the OH and HH partial RDFs.[3]

S4. On the Bonded Term in the GP Prior Mean

The importance of the bonded term in the structure factor is critical for capturing the long-range features in momentum-space. Indeed, most of the long-range oscillatory behavior observed in momentum-space arises from this component of the mean, which becomes apparent when considering the rFT of a shifted Gaussian,

$$\boldsymbol{\mathcal{H}}_r^{-1}\left[\frac{1}{\sqrt{2\pi\sigma^2}}\exp\left(-\frac{(r-r_0)^2)}{2\sigma}\right)\right] = 4\pi\rho\int_0^\infty \frac{\sin(qr)}{qr\sqrt{2\pi\sigma^2}}\exp\left(-\frac{(r-r_0)^2)}{2\sigma}\right)r^2dr. \tag{13}$$

Assuming the mean is sufficiently far from the origin and the distribution is narrow enough to contribute negligibly for $r < 0$ (which is appropriate for chemical bonds), we can extend the integration bounds to the entire real line. This transformation recasts the integral as an

expectation value,

$$\mathcal{H}_r^{-1}\left[\frac{1}{\sqrt{2\pi\sigma^2}}\exp\left(-\frac{(r-r_0)^2)}{2\sigma}\right)\right] \approx 4\pi\rho\frac{\mathbb{E}_{r\sim\mathcal{N}(r_0,\sigma^2)}\left[\sin(qr)qr\right]}{q^2}. \tag{14}$$

Next, by introducing the change of variable $y = qr$, applying Euler's identity $\mathrm{Im}[e^{iy}] = \sin(y)$, completing the square in the exponent, and identifying a new Gaussian with a complex mean, the resulting expectation yields an approximate, yet analytical, expression for the bonded portion of the structure factor mean,

$$\mu_{\mathrm{Bonded}} = \frac{4\pi\rho\exp\left(-\frac{(q\sigma)^2}{2}\right)\left((q\sigma)^2\cos(qr_0) + qr_0\sin(qr_0)\right)}{q^2}. \tag{15}$$

A closer look reveals that the dominant term decays as $\exp\left(-(q\sigma)^2\right)/q$, with the standard deviation σ controlling the decay rate. A larger σ implies a looser, more flexible bond with the central atom, suggesting that the high-q decay rate reflects the bond's strength.

S5. Visualizing Error Bars in the Posterior Distribution

The posterior function can be visualized in several ways, the most intuitive being the use of error bars around the mean value. However, care must be taken in interpreting these error bars, as they reflect distinct sources of variation in the posterior at a given point q or r.

There are two choices we can make for the error bars, both of which stem from an underlying choice of $\mathbf{\Sigma}_{\mathrm{Post}}$. The first corresponds to a *noise-free* posterior. While one might expect the standard deviation, $\boldsymbol{\sigma} = \mathrm{diag}(\mathbf{\Sigma}_{\mathrm{Post}})^{0.5}$, to satisfy the typical 68-95-99.7 rule with respect to the experimental data, this is not the case here. Since we are considering a *noise-free* posterior, the visualization does not account for statistical fluctuations arising from ω. Although the visualization does not reflect the statistical noise in the data, the underlying calculation accounts for it when conditioning on the observed data.

For this work, we have chosen to present the *noise-free* posterior, as a real-space counterpart to ω is not available. With the sources of variation established, the error bars on the posterior distribution are given by,

$$\boldsymbol{\sigma}_q = \mathrm{diag}(\boldsymbol{\Sigma}_{\mathrm{Post}})^{0.5}, \qquad \boldsymbol{\sigma}_r = \mathrm{diag}(\boldsymbol{\Sigma}_{\mathrm{Post,RDF}})^{0.5}. \tag{16}$$

S6. Coordination Number Analysis

In addition to enforcing physical constraints during uncertainty quantification, the non-stationary GP framework provides a principled foundation for calculating physical properties and their uncertainty bounds derived from the RDF, such as the coordination number.

The coordination number is the average number of type β neighbors within a distance R of a reference atom of type α, given by

$$n(R) = 4\pi\rho_\beta \int_0^R g_{\alpha,\beta}(r)\, r^2\, dr. \tag{17}$$

This standard expression depends sensitively on the choice of integration bounds, which introduces two well-known limitations in conventional scattering analyses. For the lower bound, a nonzero value of $r_{\min}$ is often selected to suppress spurious low-r features that arise from artifacts in the Fourier transform. In contrast, the physics-informed prior in our GP framework mitigates these distortions directly, removing the need for such *ad hoc* corrections. Furthermore, it has been shown that the choice of the upper bound R can significantly influence coordination number estimates.[4] Here, this source of uncertainty is explicitly accounted for by sampling from the posterior, enabling principled propagation of uncertainty into the coordination number distribution.

In all technicality, $n_{\alpha,\beta}$ is a functional operator that maps from some function space to $\mathbb{R}^+$. This motivates the use of an alternate notation $\tilde{\boldsymbol{n}}[g_{\alpha,\beta}(r); R]$ to emphasize this fact. Suppose now that you had two different RDF functions, $f(r)$ and $g(r)$, as well as two scalars a and b.

It is clear that,

$$\tilde{\boldsymbol{n}}[ag(r) + bf(r); R] = a\tilde{\boldsymbol{n}}[g(r); R] + b\tilde{\boldsymbol{n}}[f(r); R] \tag{18}$$

which implies the number distribution of particles must also be a Gaussian process due to the linearity of the transformation. This feature implies we have the distribution,

$$\tilde{\boldsymbol{n}}[g(r); R] \sim \mathcal{N}\left(m, s^2\right) \tag{19}$$

where the mean m is given by

$$m = \tilde{\boldsymbol{n}}[\mu_{\text{Post,RDF}}; R] = 4\pi\rho \int_0^R \mu_{\text{Post,RDF}} r^2 \, dr \tag{20}$$

$$= 4\pi\rho \int_0^R \left(\mu(r) + K(r, \boldsymbol{q})(\hat{\boldsymbol{K}}_{\boldsymbol{q},\boldsymbol{q}} + \omega^2 \hat{\boldsymbol{I}})^{-1}(\boldsymbol{Y} - \boldsymbol{\mu}_{\boldsymbol{q}})\right) r^2 dr \tag{21}$$

and standard deviation s is given by

$$s^2 = \tilde{\boldsymbol{n}}[\tilde{\boldsymbol{n}}[\Sigma_{\text{Post,RDF}}; R]; R] = (4\pi\rho)^2 \int_0^R \int_0^R \Sigma_{\text{Post,RDF}} \cdot (r\ r')^2 dr dr' \tag{22}$$

$$= (4\pi\rho)^2 \int_0^R \int_0^R \left(K(r, r') - K(r, \boldsymbol{q})(\hat{\boldsymbol{K}}_{\boldsymbol{q},\boldsymbol{q}} + \omega^2 \hat{\boldsymbol{I}})^{-1} K(\boldsymbol{q}, r')\right) \cdot (r\ r')^2 dr dr'. \tag{23}$$

In principle these could be computed analytically if the form of K and μ permitted, however we again opt for a numerical quadrature to not restrict the kernel design process outlined above.

Although it might be tempting to use the distribution of $\tilde{\boldsymbol{n}}[g(r); R]$ near the first minimum of the RDF to estimate the first coordination number, this approach does not produce the correct result. Note the first coordination number is given by,

$$n_1 = \tilde{\boldsymbol{n}}[g(r); r_1], \tag{24}$$

where r_1 is the location of the first local minima in $g(r)$. Due the underlying RDF being a random function, its corresponding first minima will also be a random variable. This implies that n_1 should be a mixture of Gaussians. Not only that, it really is an infinite mixture of Gaussians as there is always a non-zero probability of the RDF having a maxima on the entire support of $g(r)$ due to our assumption that the process is Gaussian. This would mean we would need to first compute the probability distribution over r_1 first, and then propagate that uncertainty through into a computation of n_1.

Unfortunately, if the structural prior is at all complicated then the estimation of the probabilities $p(r_1)$ or $p(n_1)$ may not even be analytical, clearly leading to difficulty. In this work, we resolve this issue using a Monte Carlo approach. For each sampled RDF, we identify the minimum r_1^* via a search algorithm, then evaluate eq (17) at $R = r_1^*$ to compute the corresponding coordination number. Repeating this procedure builds up a histogram of coordination numbers across the ensemble. A similar strategy may be taken to obtain any quantity of interest derived from $g(r)$ or $S(q)$.

S7. Experimental Argon Analysis Details

The $S(q)$ dataset was generated via interpolation of the reported data in Yarnell 1973.[5] Once the dataset was interpolated we added a zero mean normal noise with standard deviation 0.04 to represent approximate error due to reactor source measurements as reported in Figure 5 in the original publication.

The set of parameters in table S1 were optimized using AdamW with a batch size of 400 over the 400 available data points and a learning rate of $5 \cdot 10^{-2}$. The optimization was run for 400 epochs, with a real space integration grid spanning from a small value close to zero (0.0001) to 25, divided into 1000 evenly spaced points. The evolution of the negative log marginal Likelihood (LMLH) during the optimization process is shown in figure S1.

Table S1: Initial parameters, optimized parameters, and their bounds for experimental argon dataset.

Parameter	Initial Value	Lower Bound	Upper Bound	Optimized Value	Δ	% Change
ℓ	1.300000	0.100000	2.500000	0.921502	-0.378498	-15.77%
Max	0.800000	0.200000	4.000000	1.398309	0.598309	15.74%
Slope	25.050000	0.100000	50.000000	19.386559	-5.663441	-11.35%
Loc	4.250000	0.500000	8.000000	3.404966	-0.845034	-11.27%
Decay	0.525000	0.050000	1.000000	0.342376	-0.182624	-19.22%
σ_n	0.250050	0.000100	0.500000	0.042634	-0.207416	-41.49%
r_0	4.250000	0.500000	8.000000	3.535961	-0.714039	-9.52%
s	25.050000	0.100000	50.000000	26.542523	1.492523	2.99%

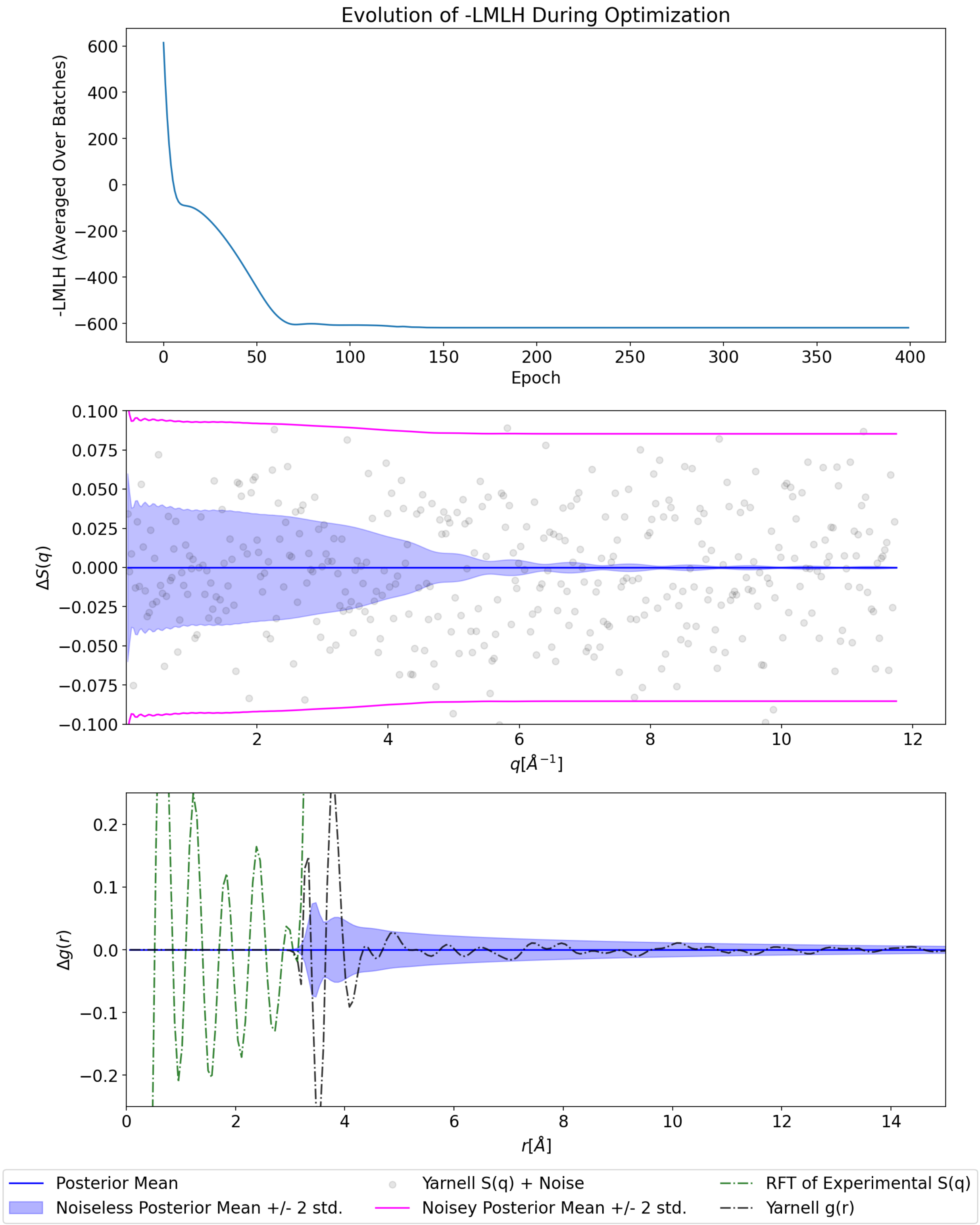

Figure S1: Top: The negative log marginal likelihood as a function of epoch during gradient descent optimization. Middle: Difference between the non-stationary GP mean and the data with noiseless and noisy posterior credibility intervals. Bottom: Argon RDF residuals for the non-stationary GP (blue), Yarnell data with direct radial Fourier Transform (green dashed line) and Yarnell data with data smoothing and regularization (black dashed line).

S8. Simulated Water Data Analysis

Molecular dynamics simulations of water were conducted using GROMACS 2023.3,[6] employing the flexible TIP4P/2005f water model.[7] The system consisted of 1500 water molecules placed in a cubic periodic simulation box at the experimental density of approximately 1 g cm^{-3}. Initial energy minimization was performed using the steepest-descent algorithm with a step size of 0.01 nm and a convergence criterion of 100 kJ mol^{-1} nm^{-1}. After minimization, the system underwent a brief 0.1 ns equilibration in the NVT ensemble at 298.15 K using the velocity-rescale thermostat[8] with a coupling constant of 1 ps. Due to the flexibility of intramolecular potentials, the simulation timestep was set to 0.2 fs. Subsequently, the system was equilibrated for an additional 1 ns under NPT conditions at 298.15 K and 1 bar. The temperature was maintained at 298.15 K using the velocity-rescale thermostat with a coupling constant of 1 ps, while pressure was regulated with an isotropic C-rescale barostat[9] employing a coupling constant of 3 ps. Electrostatic interactions were computed via the particle mesh Ewald (PME) method[10] with a cutoff of 1.0 nm, Fourier grid spacing of 0.10 nm, and fourth-order spline interpolation. Lennard-Jones interactions used a potential-shift cutoff scheme at 1.0 nm along with long-range dispersion corrections. Neighbor lists were updated every five simulation steps with the Verlet scheme, applying a buffer tolerance of 5×10^{-3} kJ mol^{-1}. Production simulation was carried out for 10 ns using the same settings as described for the NPT equilibration run. Intramolecular O-H bonds and H-O-H angles were explicitly flexible according to the TIP4P/2005f potential, and massless dummy site geometries were constrained using the LINCS algorithm of sixth order with one iteration.[11] Calculations of radial distribution functions were conducted using the Visual Molecular Dynamics (VMD) software.[12]

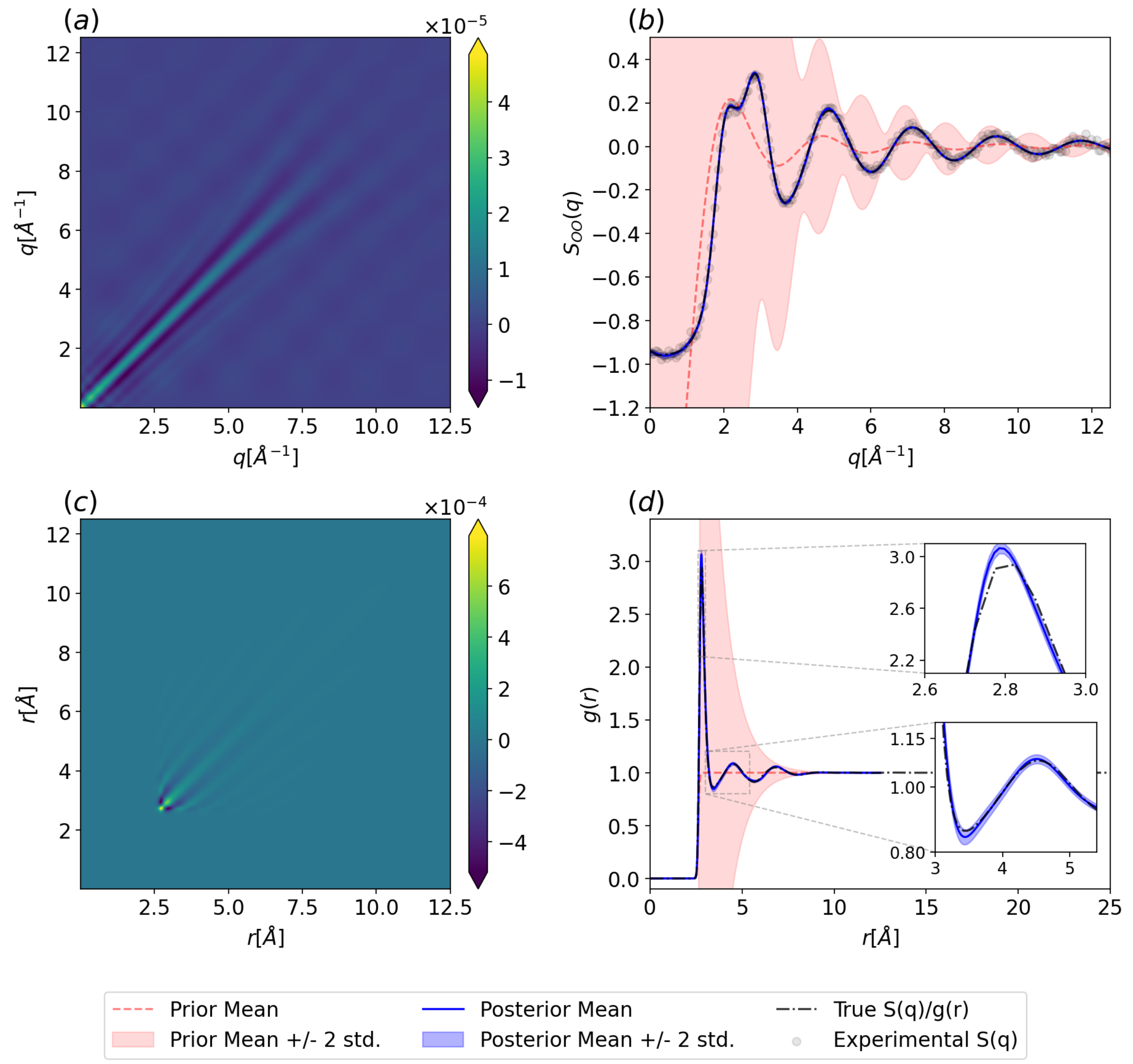

Figure S2: Posterior of the Gaussian process fit to structure factors derived from flexible TIP4P/2005f water for the oxygen-oxygen correlation.

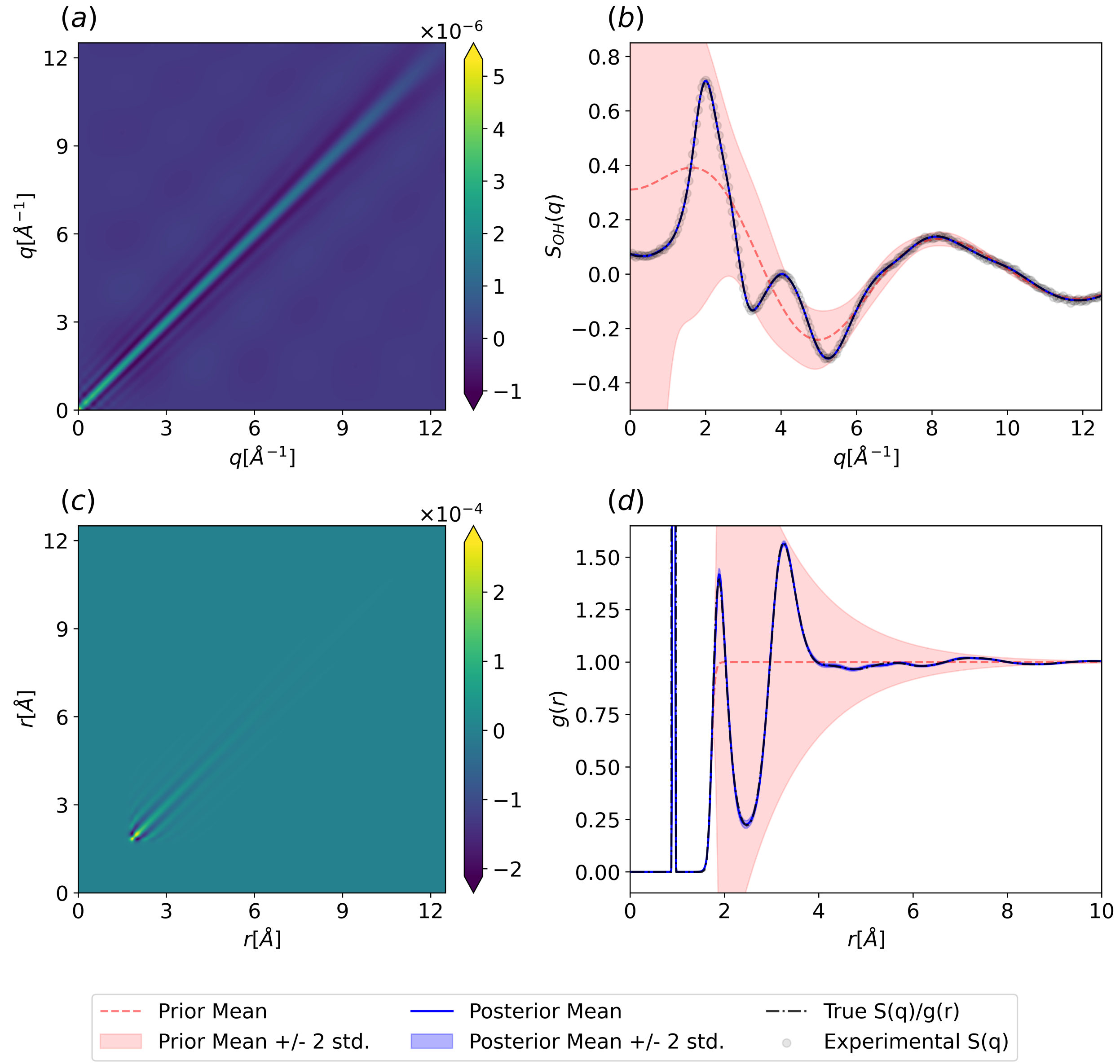

Figure S3: Posterior of the Gaussian process fit to structure factors derived from flexible TIP4P/2005f water for the oxygen-hydrogen correlation.

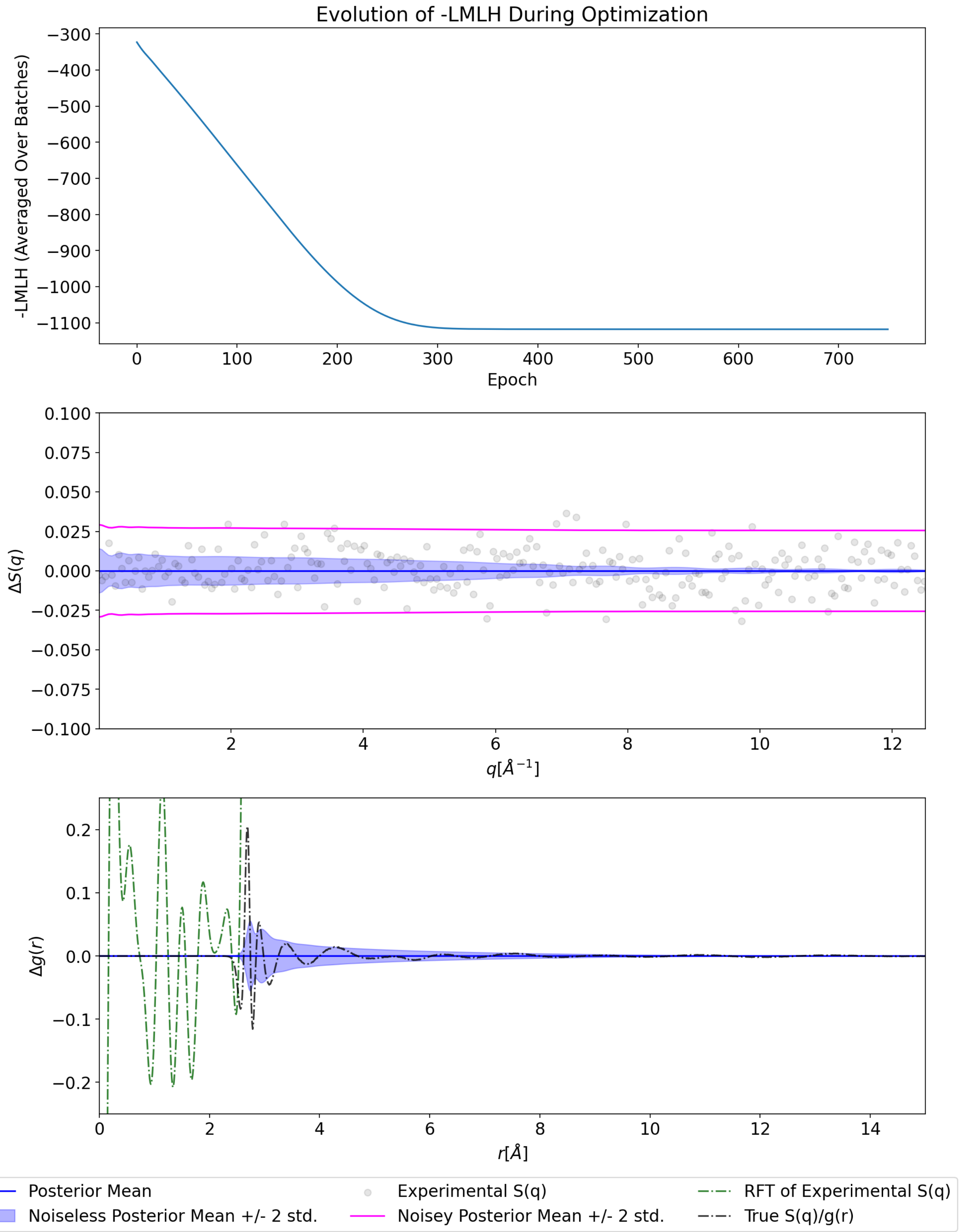

Figure S4: Top: The negative log marginal likelihood as a function of epoch during gradient descent optimization. Middle: Simulated OO structure factor residuals. Bottom: Simulated OO radial distribution function residuals.

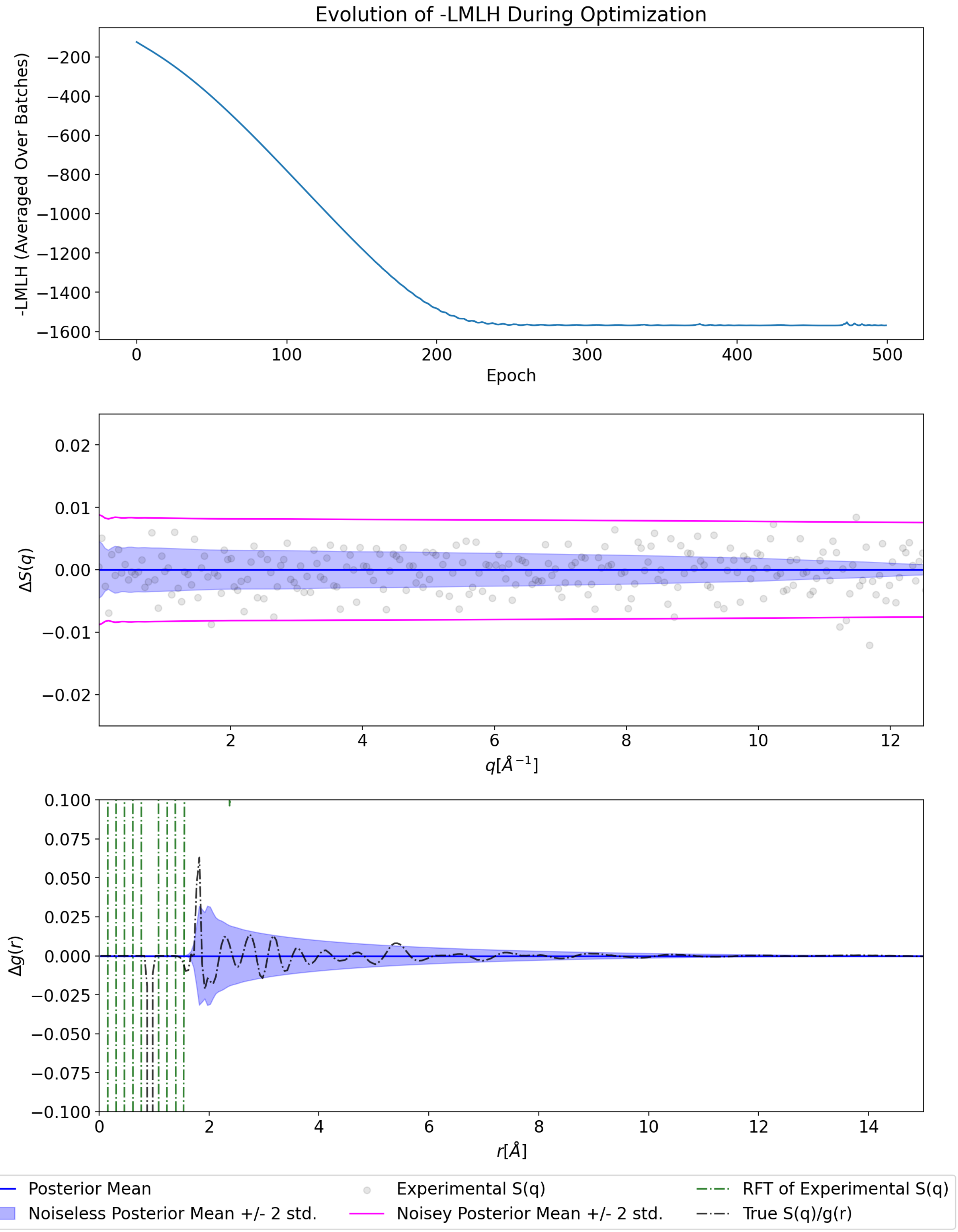

Figure S5: Top: The negative log marginal likelihood as a function of epoch during gradient descent optimization. Middle: Simulated OH structure factor residuals. Bottom: Simulated OH radial distribution function residuals.

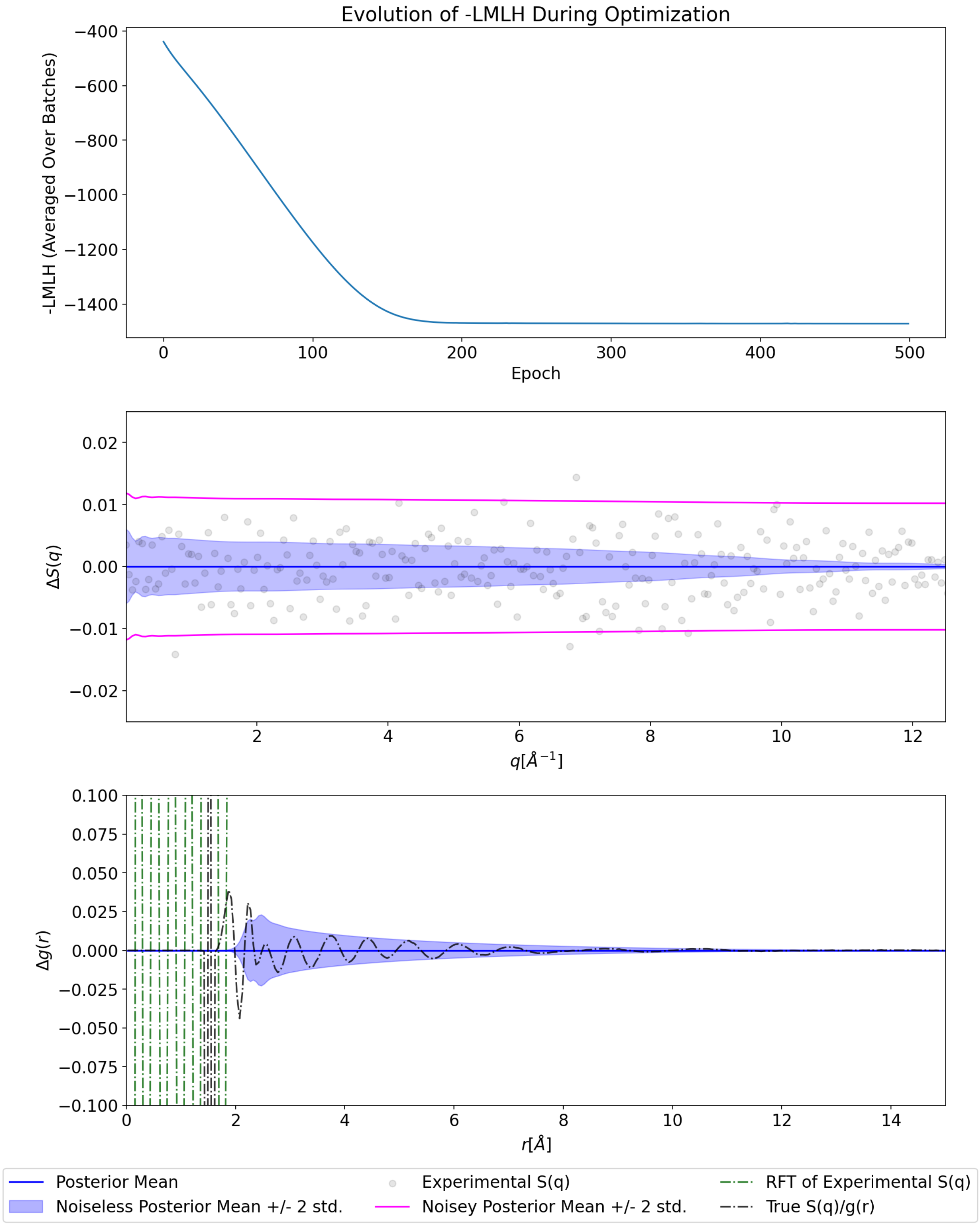

Figure S6: Top: The negative log marginal likelihood as a function of epoch during gradient descent optimization. Middle: Simulated HH structure factor residuals. Bottom: Simulated HH radial distribution function residuals.

S9. Experimental Water Analysis

Provided below are the log-marginal likelihood evolution and residuals for the oxygen-oxygen partial structure factor from the experimental X-ray scattering dataset. Optimized hyperparameters are provided in Table S2.

Table S2: Initial parameters, optimized parameters, and their bounds for experimental OO dataset.

Parameter	Initial Value	Lower Bound	Upper Bound	Optimized Value	Δ	% Change
ℓ	1.230821	0.100000	2.500000	0.808045	-0.422776	-17.62%
Max	3.000000	0.200000	10.000000	7.977876	4.977876	50.79%
Slope	5.678962	1.000000	50.000000	11.264750	5.585788	11.40%
Loc	2.948104	0.500000	6.000000	2.790726	-0.157378	-2.86%
Decay	0.534100	0.050000	3.000000	0.895524	0.361424	12.25%
σ_n	0.147792	0.000100	0.500000	0.005118	-0.142674	-28.54%
r_0	2.810502	0.010000	20.000000	2.662981	-0.147521	-0.74%
s	25.084942	0.001000	50.000000	45.947342	20.862400	41.73%

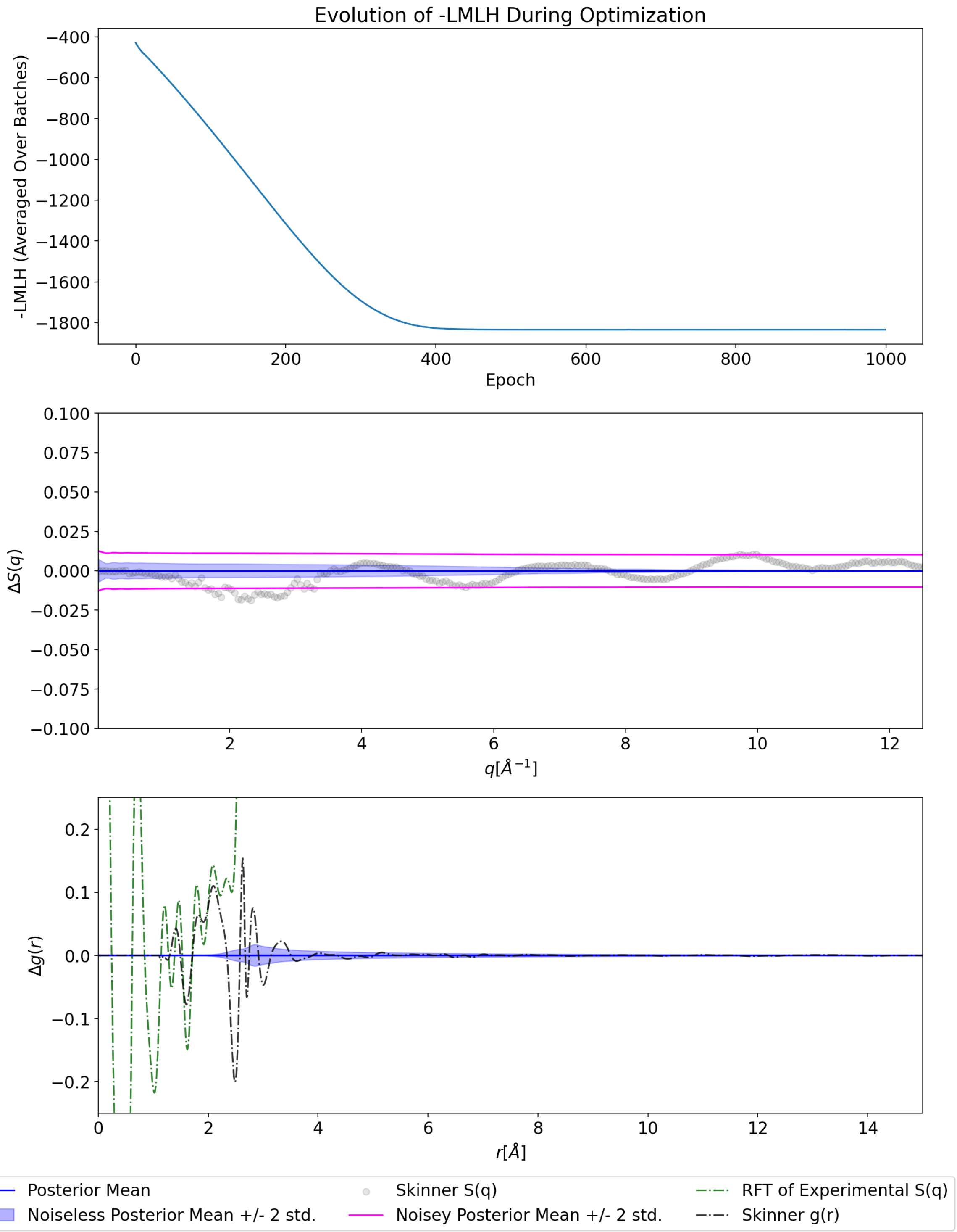

Figure S7: Top: The negative log marginal likelihood as a function of epoch during gradient descent optimization of equation 39. Middle: OO structure factor residuals. Bottom: OO radial distribution function error comparison between prior (red), posterior (dark blue) and skinners (light blue).

References

(1) Kingma, D. P.; Ba, J. Adam: A Method for Stochastic Optimization. 2017; `http://arxiv.org/abs/1412.6980`, arXiv:1412.6980 [cs].

(2) Shanks, B. L.; Potoff, J. J.; Hoepfner, M. P. Transferable force fields from experimental scattering data with machine learning assisted structure refinement. *J. Phys. Chem. Lett.* **2022**, *13*, 11512–11520.

(3) Cheng, B.; Behler, J.; Ceriotti, M. Nuclear Quantum Effects in Water at the Triple Point: Using Theory as a Link Between Experiments. *J. Phys. Chem. Lett.* **2016**, *7*, 2210–2215.

(4) Skinner, L. B.; Huang, C.; Schlesinger, D.; Pettersson, L. G. M.; Nilsson, A.; Benmore, C. J. Benchmark oxygen-oxygen pair-distribution function of ambient water from x-ray diffraction measurements with a wide Q-range. *J. Chem. Phys.* **2013**, *138*, 074506.

(5) Yarnell, J. L.; Katz, M. J.; Wenzel, R. G.; Koenig, S. H. Structure factor and radial distribution function for liquid argon at 85K. *Phys. Rev. A* **1973**, *7*, 2130–2144.

(6) Abraham, M. J.; Murtola, T.; Schulz, R.; Páll, S.; Smith, J. C.; Hess, B.; Lindahl, E. GROMACS: High performance molecular simulations through multi-level parallelism from laptops to supercomputers. *SoftwareX* **2015**, *1-2*, 19–25.

(7) González, M. A.; Abascal, J. L. F. A flexible model for water based on TIP4P/2005. *J. Chem. Phys.* **2011**, *135*, 224516.

(8) Bussi, G.; Donadio, D.; Parrinello, M. Canonical sampling through velocity rescaling. *J. Chem. Phys.* **2007**, *126*, 014101.

(9) Bernetti, M.; Bussi, G. Pressure control using stochastic cell rescaling. *J. Chem. Phys.* **2020**, *153*, 114107.

(10) Essmann, U.; Perera, L.; Berkowitz, M. L.; Darden, T.; Lee, H.; Pedersen, L. G. A smooth particle mesh Ewald method. *J. Chem. Phys.* **1995**, *103*, 8577–8593.

(11) Hess, B.; Bekker, H.; Berendsen, H. J. C.; Fraaije, J. G. E. M. LINCS: A linear constraint solver for molecular simulations. *J. Comput. Chem.* **1997**, *18*, 1463–1472.

(12) Humphrey, W.; Dalke, A.; Schulten, K. VMD: Visual molecular dynamics. *J. Mol. Graph.* **1996**, *14*, 33–38.
